# Effects of Curcumin Supplementation on Inflammatory Biomarkers in Adults with Type 2 Diabetes Mellitus: A Systematic Review and Meta-Analysis of Randomized Controlled Trials

**DOI:** 10.3390/nu18183028

**Published:** 2026-09-16

**Authors:** Claudio Ignacio Pérez, Jorge Enrique González-Casanova, Diana Marcela Rojas-Gómez

**Affiliations:** 1 Escuela de Nutrición y Dietética, Facultad de Medicina, Universidad Andres Bello, Santiago 8370321, Chile; c.prezprez2@uandresbello.edu; 2 Instituto de Ciencias Biomédicas, Facultad de Ciencias de la Salud, Universidad Autónoma de Chile, Santiago 8910060, Chile

**Keywords:** curcumin, type 2 diabetes mellitus, inflammation, hs-CRP, TNF-α, systematic review, meta-analysis, randomized controlled trial

## Abstract

**Background/Objectives:** Chronic low-grade inflammation contributes to the pathophysiology of type 2 diabetes mellitus (T2DM). This systematic review and meta-analysis evaluated randomized evidence on the effects of oral curcumin or curcuminoid supplementation on circulating inflammatory biomarkers in adults with T2DM. **Methods:** PubMed, Scopus, and Web of Science were searched from database inception through 31 August 2026. Random-effects models using restricted maximum likelihood (REML) and Hartung–Knapp confidence intervals were applied to the primary syntheses because relatively few studies contributed to each outcome. **Results:** Six randomized controlled trials (RCTs; *n* = 472) provided compatible hs-CRP endpoint data. Curcumin supplementation was associated with lower hs-CRP concentrations (MD = −1.06 mg/L; 95% CI −1.97 to −0.16; *p* = 0.030; *I*^2^ = 17.3%), although sensitivity analyses involving unit-ambiguous or statistically reconstructed data increased uncertainty. Five RCTs (*n* = 475) contributed quantitative TNF-α data; the pooled standardized effect favored curcumin (Hedges’ g = −1.20; 95% CI −1.98 to −0.42; *p* = 0.013), with substantial heterogeneity (*I*^2^ = 84.9%). Evidence for IL-6 from three randomized cohorts was directionally favorable but heterogeneous and was synthesized narratively, whereas IL-1β findings were limited to a single randomized cohort. **Conclusions:** Curcumin or curcuminoid supplementation may reduce hs-CRP and TNF-α in adults with T2DM; however, the strength of inference is limited by the small evidence base, heterogeneity, and inconsistencies in outcome reporting. Larger, methodologically standardized trials are warranted.

## 1. Introduction

Type 2 diabetes mellitus (T2DM) is a major contributor to the global burden of chronic disease and is closely linked to cardiovascular, renal, and metabolic morbidity. In 2024, approximately 589 million adults aged 20–79 years worldwide were living with diabetes, and this number is projected to increase to approximately 853 million by 2050. T2DM accounts for over 90% of diabetes cases [1]. Although impaired insulin action, progressive pancreatic β-cell dysfunction, and persistent hyperglycemia remain central features of the disease, T2DM is increasingly understood as a disorder in which metabolic abnormalities coexist with sustained activation of inflammatory pathways [2,3,4].

This chronic, low-grade inflammatory state is closely connected to adipose-tissue dysfunction and altered immune signaling. Expansion of visceral adipose tissue promotes the recruitment and activation of macrophages and other innate immune cells, increasing the production of pro-inflammatory mediators such as tumor necrosis factor-alpha (TNF-α), interleukin-6 (IL-6), and interleukin-1 beta (IL-1β) [5,6]. These mediators can interfere with insulin signaling and activate intracellular pathways including nuclear factor kappa B (NF-κB) and mitogen-activated protein kinases (MAPKs), thereby contributing to insulin resistance, oxidative stress, endothelial dysfunction, impaired glucose homeostasis, and progressive β-cell injury [3,7].

Circulating inflammatory biomarkers therefore provide clinically relevant information regarding the inflammatory component of T2DM. High-sensitivity C-reactive protein (hs-CRP), TNF-α, IL-6, and IL-1β are among the markers most frequently investigated in this population. Higher concentrations have been associated with poorer metabolic control, greater insulin resistance, vascular dysfunction, and increased cardiovascular risk [8,9,10]. hs-CRP is particularly useful as an indicator of systemic low-grade inflammation and has also been associated with cardiovascular risk [10,11]. In parallel, TNF-α, IL-6, and IL-1β participate directly in pathways involved in insulin resistance, β-cell dysfunction, and vascular injury [3]. These observations provide a rationale for evaluating nutritional interventions capable of modulating inflammatory activity in adults with T2DM.

Among dietary bioactive compounds, curcumin has attracted considerable interest because of its potential effects on inflammatory and oxidative pathways. Curcumin is the principal polyphenolic constituent of *Curcuma longa* and has been investigated for antioxidant, anti-inflammatory, metabolic, and cardiovascular effects [12,13,14,15]. Experimental evidence indicates that curcumin can interfere with NF-κB activation, reduce the expression of pro-inflammatory cytokines, influence MAPK-related signaling, and stimulate nuclear factor erythroid 2-related factor 2 (Nrf2)-dependent antioxidant responses [12,14,16,17]. Activation of Nrf2 may enhance endogenous antioxidant defenses involving superoxide dismutase, glutathione peroxidase, and catalase [12,16,17]. These mechanisms provide biological plausibility for a potential effect of curcumin on systemic inflammatory biomarkers in T2DM.

Despite this mechanistic rationale, clinical evidence remains difficult to interpret. Previous systematic reviews and meta-analyses have generally reported favorable effects of curcumin or turmeric supplementation on inflammatory outcomes, including CRP, IL-6, and TNF-α [18,19]. However, many of these syntheses combined participants with different clinical conditions, limiting their applicability specifically to adults with established T2DM. T2DM-focused analyses have also produced divergent findings. Liang [20] reported no statistically significant effect on hs-CRP and substantial heterogeneity, whereas Mokgalaboni et al. [21] observed a significant reduction in CRP but similarly identified considerable between-study variability. More recently, Bahari et al. [22] reported reductions in CRP, TNF-α, and IL-6, although that review included both prediabetes and T2DM and combined inflammatory and oxidative-stress outcomes.

Several methodological factors may explain these differences across previous syntheses. Eligible populations have not always been equivalent, curcumin formulations and doses vary substantially, inflammatory biomarkers have been reported using different analytical methods and statistical formats, and individual randomized trials have not been consistently incorporated across reviews. In addition, the distinction between CRP and hs-CRP, the handling of converted summary statistics, and the use of different effect measures can materially influence quantitative estimates. Consequently, uncertainty remains regarding both the magnitude and the consistency of the anti-inflammatory effect of curcumin specifically in adults with established T2DM.

The present systematic review and meta-analysis therefore evaluated randomized controlled trials of oral curcumin or standardized curcuminoid preparations in adults with established T2DM, without restriction by publication year. The search strategy was broadened to maximize sensitivity and capture older randomized evidence that could be missed by recent-evidence-only approaches. hs-CRP was synthesized using mean differences when values could be expressed in clinically compatible units, whereas TNF-α was analyzed using standardized mean differences because of variation in outcome distributions and measurement scales across trials. IL-6 and IL-1β were synthesized narratively when the available evidence did not support a stable quantitative summary. Risk of bias was assessed using the Cochrane RoB 2 tool, and certainty of evidence was evaluated using the GRADE framework. The objective was to determine the magnitude, consistency, and certainty of the effects of curcumin supplementation on circulating inflammatory biomarkers in adults with T2DM.

## 2. Materials and Methods

### 2.1. Study Design and Protocol

This systematic review and meta-analysis was conducted in accordance with the PRISMA 2020 statement. Its primary objective was to evaluate the effects of oral curcumin supplementation on circulating inflammatory biomarkers in adults with T2DM, with particular emphasis on hs-CRP, TNF-α, IL-6, and IL-1β.

The review protocol was developed a priori in accordance with recommendations from the Cochrane Handbook [23], but it was neither prospectively registered nor publicly archived. This manuscript and the Supplementary Information document the review methods and subsequent amendments, including expansion of the search to database inception. All stages of the review, including literature searching, study selection, data extraction, risk-of-bias assessment, statistical analysis, and certainty-of-evidence evaluation, followed predefined methodological criteria.

Only randomized controlled trials (RCTs) comparing oral curcumin or curcuminoid supplementation with placebo or standard care in adults with T2DM were eligible.

### 2.2. Eligibility Criteria (PICOS)

The core eligibility criteria were defined a priori according to the Population, Intervention, Comparator, Outcomes, and Study design (PICOS) framework. The original publication-date restriction was subsequently removed as a documented methodological amendment when the literature search was expanded to database inception (Table 1).

Population (P): Studies enrolling adults (≥18 years) with type 2 diabetes mellitus (T2DM), regardless of sex, ethnicity, body mass index, disease duration, or concomitant pharmacological treatment, were considered eligible. Studies including participants with type 1 diabetes mellitus, gestational diabetes, prediabetes, or other specific forms of diabetes were excluded.

Intervention (I): Eligible studies were randomized controlled trials evaluating oral curcumin or standardized curcuminoid supplementation, including nano-curcumin, nanomicellar curcumin, curcuminoid extracts, phytosomal preparations, or other formulations with defined curcumin or curcuminoid content, irrespective of dosage or intervention duration. Studies using whole turmeric powder or turmeric rhizome without defined curcumin or curcuminoid content were excluded. Studies evaluating curcumin in combination with another active compound were included only when the effect of the curcumin-containing intervention could be independently assessed. Piperine or black pepper used solely as a bioavailability-enhancing component of a curcumin formulation was not treated as an independent therapeutic co-intervention.

Comparator (C): Eligible studies compared curcumin supplementation with placebo or standard care. Trials comparing different curcumin formulations without a placebo or control group were excluded.

Outcomes (O): Outcomes of interest were post-intervention circulating concentrations of high-sensitivity C-reactive protein (hs-CRP), tumor necrosis factor-α (TNF-α), interleukin-6 (IL-6), and interleukin-1β (IL-1β). hs-CRP was prespecified as the primary outcome, whereas TNF-α, IL-6, and IL-1β were secondary outcomes. Studies reporting at least one eligible biomarker with sufficient quantitative data (mean and standard deviation or convertible statistics) were included in the corresponding quantitative synthesis when the data were sufficiently comparable. Biomarkers reported in only one eligible study or without comparable statistical information were summarized narratively.

Study design (S): Only randomized controlled trials (parallel or crossover design) were eligible. Primary RCT reports were required to be full-text articles published in peer-reviewed journals. Observational studies, case reports, case series, conference abstracts, review articles, editorials, letters without original trial data, study protocols, animal studies, and in vitro investigations were excluded. Secondary or companion reports arising from an otherwise eligible randomized controlled trial were retained when they provided original, extractable data for a prespecified outcome and could be unequivocally linked to the parent trial.

No restrictions on publication year or geographical location were applied in the final updated search. The expanded search addressed the limitations of the earlier recent-evidence-only window and enabled an inception-based assessment of the randomized evidence.

### 2.3. Literature Search Strategy

The final update used a sensitivity-first search strategy based on two concepts—curcumin/curcuminoids and type 2 diabetes mellitus—without requiring an inflammatory-biomarker term. This broader strategy was intended to identify trials in which inflammatory outcomes were secondary or incompletely indexed. Appendix A provides reproducible database-specific syntax for the locked search concept, and the review audit files retain the corresponding export counts and search documentation.

PubMed, Scopus, and the Web of Science Core Collection were searched from database inception through 31 August 2026. The final exports included 219 records from PubMed, 465 from Scopus, and 577 from Web of Science, totaling 1261 records before cross-database deduplication. Reference lists of eligible trials and relevant reviews were also examined for additional potentially eligible reports, which were tracked separately as records identified through other methods.

The Cochrane Central Register of Controlled Trials (CENTRAL) and Ovid MEDLINE were not included in the final search update because complete institutional access and/or export functionality was unavailable. To maximize retrieval sensitivity within the available resources, the final search used broad inception-based strategies without a mandatory inflammatory-biomarker term, and reference lists of eligible trials and relevant reviews were additionally examined.

The expanded search removed the previous 2020–2025 date restriction. Final reporting distinguishes completed inception database searches from citation-chasing records and does not retrospectively attribute records identified through other methods to the electronic database count.

### 2.4. Study Selection

Records retrieved from the electronic databases were imported into reference-management software and deduplicated across sources. After duplicate removal, unique records were transferred to Rayyan QCRI (Rayyan Systems Inc., Cambridge, MA, USA; web platform; accessed 31 August 2026) for organization and documentation of the screening process.

Two reviewers independently assessed study eligibility in two sequential stages. First, titles and abstracts were screened against the prespecified criteria to identify records requiring full-text assessment. The same reviewers then independently assessed the corresponding full-text articles and determined final eligibility. Disagreements regarding study eligibility were resolved through discussion and consensus; when necessary, a third reviewer was consulted.

**Figure 1:**
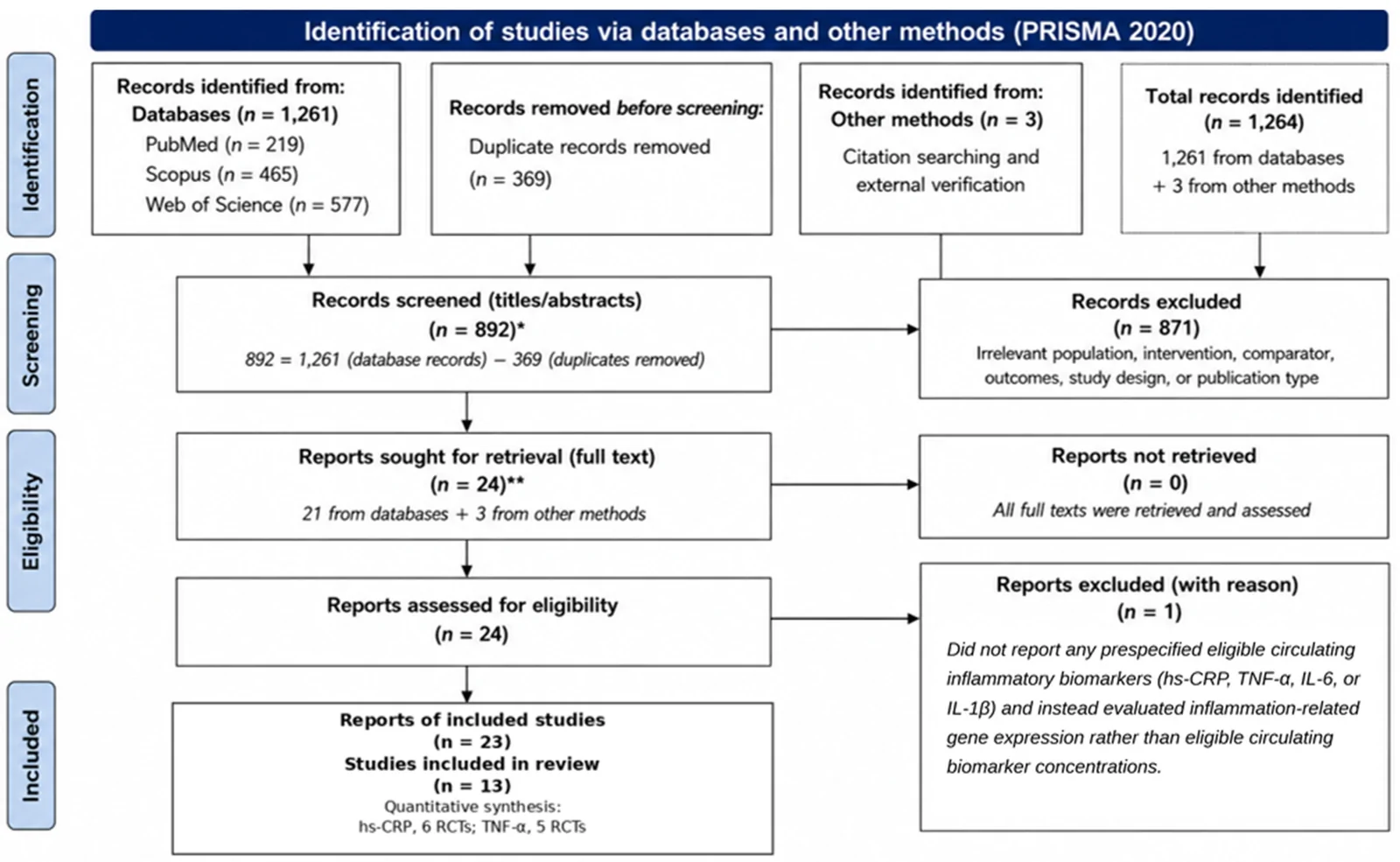
PRISMA 2020 flow diagram for the expanded inception search [24]. Database searches identified 1261 records; after removal of 369 duplicates, 892 unique records were screened and 871 were excluded. Twenty-one reports from database searches and three reports identified through other methods were assessed for eligibility. One report, by Shafabakhsh et al. [25], was excluded at full-text assessment because it evaluated inflammation-related gene expression rather than any of the prespecified eligible circulating inflammatory biomarkers (hs-CRP, TNF-α, IL-6, or IL-1β). The remaining 23 reports represented 13 independent randomized controlled trials after linked or overlapping publications were mapped to their corresponding trial families. * After remove of duplicates. ** 21 reports from databases + 3 reports from other methods.

The complete study selection process, including the numbers of records identified, screened, excluded, and included, is presented in the PRISMA 2020 flow diagram [24] (Figure 1).

### 2.5. Data Extraction

Study data were extracted independently by two reviewers using a standardized review-specific form. The two extraction datasets were compared, and discrepancies were resolved by consensus; unresolved differences were referred to a third reviewer.

For each eligible trial, extracted information included trial registration, first author, publication year, country, study design, randomized and analyzed sample sizes, participant characteristics, curcumin formulation, dosage, intervention duration, comparator, inflammatory biomarkers, laboratory methods, assessment time point, and quantitative outcome data. Publications arising from the same randomized cohort were mapped to a common trial identifier before quantitative synthesis.

For continuous outcomes, the preferred inputs were analyzed group sizes and post-intervention means with standard deviations. When only medians and interquartile ranges, or means accompanied by minimum and maximum values, were reported, means and dispersion measures were estimated using the method of Wan et al. [26] only when the resulting values represented the same unadjusted endpoint contrast required for pooling. All such transformations were identified in the extraction dataset and examined in sensitivity analyses. Adjusted effects reported without compatible group-level endpoints were retained separately and evaluated through generic inverse-variance sensitivity analyses rather than combined with unadjusted contrasts. Reports arising from the same randomized cohort were assigned a common trial identifier to prevent double counting of participants.

Mansour et al. [27] was excluded from the primary hs-CRP synthesis because of a baseline hs-CRP imbalance and the use of baseline-adjusted ANCOVA without reporting the adjusted treatment effect and standard error required for replication. Panahi et al. [28] was not incorporated directly into the primary hs-CRP mean-difference pool because the article reported hs-CRP in a physiologically implausible unit; this trial was instead evaluated in a unit-invariant standardized-mean-difference sensitivity analysis. The hemodialysis trial by Shafabakhsh et al. [29] reported a baseline-adjusted hs-CRP effect and was retained for a separate adjusted-effect sensitivity analysis.

When multiple publications arose from the same randomized controlled trial, only the publication providing the most complete and relevant dataset for each inflammatory biomarker was included in the quantitative synthesis to prevent duplicate inclusion of participants. Additional publications from the same trial were used only to complement the narrative synthesis when appropriate.

All extracted data were compiled and verified in Microsoft Excel before statistical analysis.

### 2.6. Risk of Bias Assessment

Two reviewers independently assessed risk of bias for the included inflammatory outcomes using the Cochrane Risk of Bias 2 (RoB 2) tool [30]. Five domains were evaluated: bias arising from the randomization process, bias due to deviations from intended interventions, bias due to missing outcome data, bias in outcome measurement, and bias in selection of the reported result [30].

**Figure 2:**
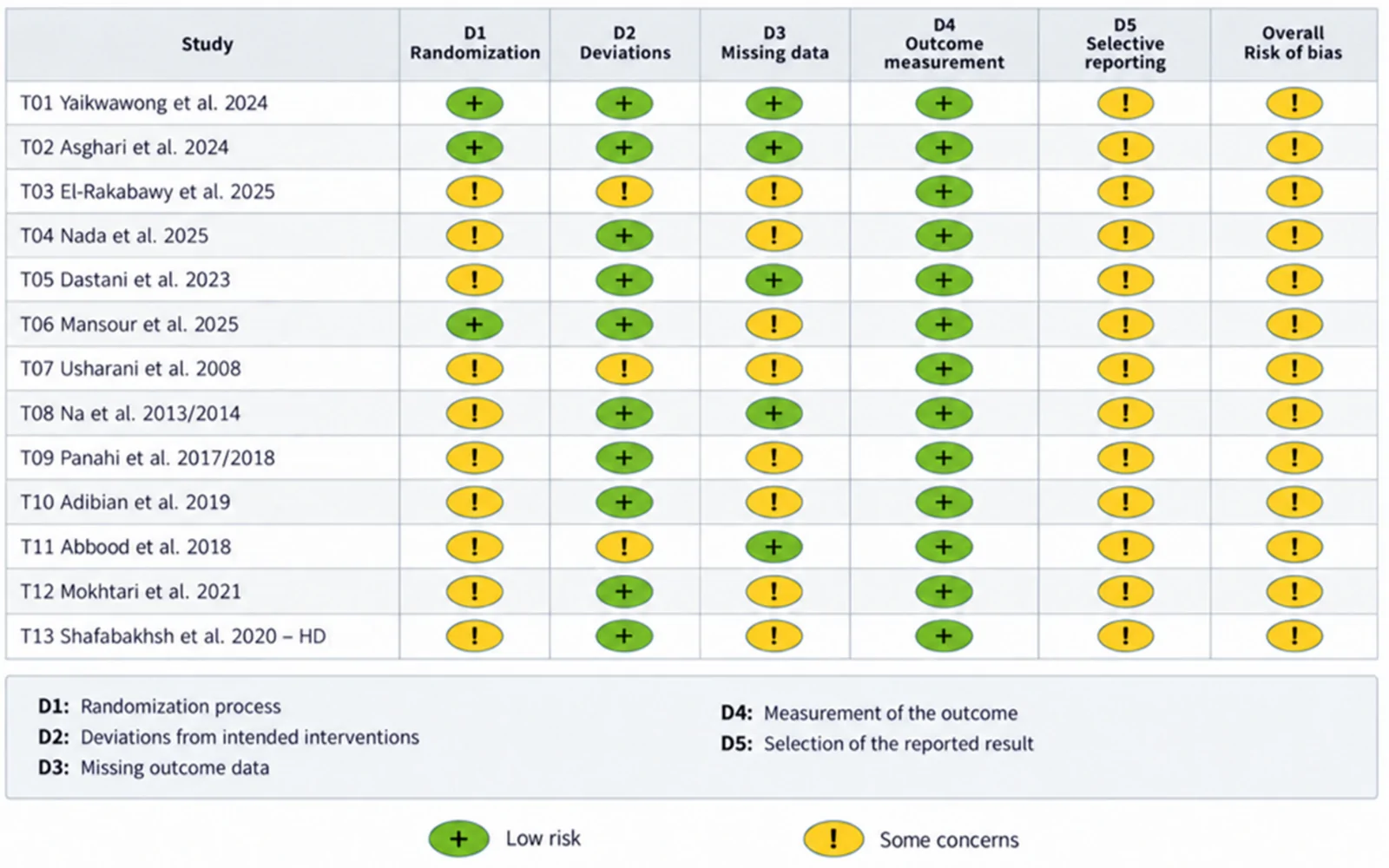
Cochrane Risk of Bias 2 (RoB 2) summary for prespecified representative inflammatory results from the 13 independent randomized controlled trials included in the systematic review. Overall, all 13 displayed results were judged to have some concerns, and none were judged at high risk of bias. The specific result represented for each trial is reported in Supplementary Table S6. Excluded studies are not displayed. D1, randomization process; D2, deviations from intended interventions; D3, missing outcome data; D4, measurement of the outcome; D5, selection of the reported result. Green circles (+) indicate low risk of bias; yellow circles (!) indicate some concerns. HD, hemodialysis. Studies shown, from top to bottom: Yaikwawong et al. [31], Asghari et al. [32], El-Rakabawy et al. [33], Nada et al. [34], Dastani et al. [35], Mansour et al. [27], Usharani et al. [36], Na et al. [37,38], Panahi et al. [28,39], Adibian et al. [40], Abbood [41], Mokhtari et al. [42], and Shafabakhsh et al. (hemodialysis trial) [29].

According to the RoB 2 algorithm, each domain was rated as low risk, some concerns, or high risk of bias. Judgments were made for a specified inflammatory result rather than as a general study-quality score. For trials reporting more than one eligible biomarker, Figure 2 presents one prespecified representative inflammatory result per trial; the result represented for each trial and the supporting domain-level rationale are reported in Supplementary Table S6. These representative rows should not be interpreted as study-wide quality ratings. Disagreements were resolved through discussion and, when necessary, consultation with a third reviewer [30].

### 2.7. Outcomes

The primary outcome was the effect of oral curcumin supplementation on circulating hs-CRP concentrations in adults with T2DM.

Secondary outcomes included changes in TNF-α, IL-6, and IL-1β. Quantitative meta-analyses were performed when at least two studies reported comparable data for a given biomarker. Biomarkers reported in only one eligible study or without sufficiently comparable quantitative information were summarized narratively.

### 2.8. Statistical Analysis

Quantitative syntheses were conducted using inverse-variance random-effects models, with between-study variance (τ^2^) estimated by restricted maximum likelihood (REML). hs-CRP was pooled as an endpoint mean difference when trial values could be expressed on the common mg/L scale; negative estimates favored curcumin. TNF-α was analyzed on a standardized scale using Hedges’ small-sample correction because the contributing trials differed materially in outcome distributions and effective measurement scales despite reporting nominally similar units.

Because relatively few independent trials contributed to each primary meta-analysis, uncertainty around the pooled hs-CRP and TNF-α effects was quantified using Hartung–Knapp-adjusted 95% confidence intervals; conventional Wald intervals were retained as sensitivity analyses. Additional hs-CRP robustness analyses included restriction to trials with directly reported endpoint means and standard deviations, leave-one-out recalculation, a unit-invariant standardized-mean-difference analysis incorporating Panahi et al. [28], and an exploratory generic inverse-variance analysis incorporating the adjusted hs-CRP estimate from the hemodialysis trial by Shafabakhsh et al. [29].

Statistical heterogeneity was assessed using Cochran’s Q test, τ^2^, and the *I*^2^ statistic. Formal funnel-plot asymmetry testing was not undertaken because fewer than ten independent trials contributed to each quantitative synthesis [43]. The final reproducible analysis workflow was implemented in R using the metafor package. Outcome-level extraction and data-preparation details are reported in Supplementary Table S5. Individual-study effects were displayed in forest plots, whereas study characteristics, sensitivity analyses, risk-of-bias judgments, and certainty assessments were summarized in structured tables in the main manuscript and Supplementary Tables S4–S8.

### 2.9. Certainty of Evidence (GRADE)

The certainty of evidence for each quantitative outcome was evaluated using the Grading of Recommendations Assessment, Development and Evaluation (GRADE) approach [44]. Evidence from randomized controlled trials was initially rated as high certainty and was then assessed across five domains: risk of bias, inconsistency, indirectness, imprecision, and publication bias. The certainty of evidence for each outcome was classified as high, moderate, low, or very low. Final GRADE judgments were determined after completion of the RoB 2 assessment and are reported with explicit downgrade rationales in the Summary of Findings (SoF) table.

## 3. Results

### 3.1. Study Selection

The database searches identified 1261 records: 219 from PubMed, 465 from Scopus, and 577 from Web of Science. After removal of 369 duplicate records, 892 unique records underwent title and abstract screening. Of these, 871 were excluded, leaving 21 reports for full-text retrieval and eligibility assessment. Three additional reports were identified through citation searching and external verification. All 24 reports were successfully retrieved and assessed for eligibility. One report, Shafabakhsh et al. [25] (T2DM + CHD), was excluded at full-text assessment, leaving 23 included reports representing 13 independent randomized controlled trials after publications arising from the same randomized cohort were linked to a common trial identifier.

The included evidence base contributed data on hs-CRP, TNF-α, IL-6, and IL-1β, with several publications representing overlapping reports from the same randomized cohort. These publications were mapped to common trial identifiers to avoid double counting of participants.

One report by Shafabakhsh et al. [25] was excluded at full-text assessment because it did not report any of the prespecified eligible circulating inflammatory biomarkers (hs-CRP, TNF-α, IL-6, or IL-1β). Instead, the study evaluated inflammation-related gene expression rather than eligible circulating biomarker concentrations.

The complete study selection process, including the numbers of records identified, screened, excluded, and included, is presented in the PRISMA 2020 flow diagram (Figure 1), in accordance with the PRISMA 2020 reporting guidelines [24].

### 3.2. Characteristics of the Included Studies

The main characteristics of the 13 independent randomized controlled trials included in the expanded inception-search review are summarized in Table 2. The evidence base spans Thailand, Iran, Egypt, India, China, and Iraq and includes conventional curcumin, standardized curcuminoid extracts, nano/nanomicellar formulations, and curcuminoids co-administered with piperine. Intervention durations ranged from 8 weeks to 12 months.

Outcome-specific quantitative syntheses were based on independent trial cohorts rather than publication counts. The primary hs-CRP analysis included six RCTs with compatible endpoint data (*n* = 472), whereas five RCTs provided group-level data for the TNF-α meta-analysis (*n* = 475). A sixth eligible TNF-α trial (Panahi et al. [28,39]) was retained narratively because the accessible report did not provide the group-level TNF-α values required for an auditable standardized effect. Group-level numerical IL-6 data were available from three independent randomized cohorts, but IL-6 was retained for narrative synthesis because of the small evidence base and substantial clinical and methodological heterogeneity (Supplementary Table S5, Panel C). IL-1β remained supported by one randomized cohort. The 2026 Yaikwawong inflammatory/NLR report [45] was mapped to the same TCTR20140303003 randomized cohort and was not treated as an additional independent RCT.

The Yaikwawong/TCTR20140303003 trial family also included companion reports on beta-cell function [46], liver steatosis [47], and depression [48]. Additional reports on antioxidant effects [49] and lipid profiles [50] were mapped to the Panahi/IRCT201505301165N4 trial family. The report on anthropometric indices, insulin resistance, and oxidative stress [51] was mapped to the Adibian/Hodaei cohort. These companion publications did not increase the number of independent randomized trials (Supplementary Tables S2 and S3).

Clinical and methodological variability was evident in curcumin formulation, dosage, background treatment, intervention duration, participant comorbidity, and biomarker reporting. These differences were explicitly considered in the choice of effect measure, sensitivity analyses, and certainty-of-evidence assessment.

**Table 2:** Characteristics of the 13 independent randomized controlled trials included in the expanded inception-search systematic review. I, intervention; C, comparator; hs-CRP, high-sensitivity C-reactive protein; TNF-α, tumor necrosis factor-α; IL, interleukin; HD, hemodialysis. Publications arising from the same randomized cohort were mapped to a common trial identifier and were not counted as independent studies. The 2026 Yaikwawong inflammatory/NLR publication [45] was mapped to TCTR20140303003 and therefore did not increase the independent-trial count. * Sample sizes are analyzed participants for the relevant comparison where available; for the Shafabakhsh hemodialysis trial, 30/30 randomized participants are shown because compatible group-level hs-CRP endpoint data were not verified for the primary mean-difference synthesis. IQR, interquartile range; SMD, standardized mean difference.

Independent Trial/Report Used	Country	Sample I/C *	Intervention and Dose	Duration	Comparator	Inflammatory Outcomes/Quantitative Role
Yaikwawong/TCTR20140303003 (2024–2026 linked reports) [31,45,52]	Thailand	113/114	Curcuminoid extract, 1500 mg/day	12 months	Placebo	hs-CRP and TNF-α quantitative; IL-6 and IL-1β narrative
Asghari et al. (2024) [32]	Iran	24/23	Nano-curcumin, 80 mg/day + omega-3 placebos	12 weeks	Double placebo	hs-CRP quantitative
El-Rakabawy et al. (2025) [33]	Egypt	36/36	Curcumin, 1500 mg/day + conventional therapy	14 weeks	Conventional therapy	TNF-α quantitative
Nada et al. (2025) [34]	Egypt	20/20	Curcumin 1100 mg + black pepper 5 mg/day + glimepiride	12 weeks	Placebo + glimepiride	hs-CRP quantitative after median/IQR conversion
Dastani et al. (2023) [35]	Iran	32/32	Nanomicellar curcumin, 80 mg/day	90 days	Placebo	hs-CRP quantitative
Mansour et al. (2025) [27]	Iran	41/45	Nanocurcumin, 80 mg/day	16 weeks	Placebo	hs-CRP exploratory sensitivity only
Usharani et al. (2008) [36]	India	23/21	NCB-02 standardized curcuminoids, 600 mg/day	8 weeks	Placebo	TNF-α quantitative; IL-6 narrative; atorvastatin arm excluded
Na et al. (2013/2014) [37,38]	China	50/50	Curcuminoids, 300 mg/day	3 months	Placebo	TNF-α quantitative; IL-6 narrative (ANCOVA-adjusted inference)
Panahi/IRCT201505301165N4 (2017/2018) [28,39]	Iran	50/50	Curcuminoids 500 mg/day + piperine 5 mg/day; linked reports	12 weeks	Placebo	hs-CRP SMD sensitivity; TNF-α narrative; overlapping publications counted once
Adibian et al. (2019) [40]	Iran	21/23	Curcumin, 1500 mg/day	10 weeks	Placebo	hs-CRP quantitative
Abbood (2018) [41]	Iraq	16/16	Curcumin, 1000 mg/day	12 weeks	Placebo	TNF-α quantitative; atorvastatin arm excluded
Mokhtari et al. (2021) [42]	Iran	25/25	Nanocurcumin, 80 mg/day	12 weeks	Placebo	hs-CRP quantitative
Shafabakhsh et al. (2020b; diabetes on HD) [29]	Iran	30/30 randomized	Nanocurcumin, 80 mg/day	12 weeks	Placebo	hs-CRP adjusted-effect sensitivity

### 3.3. Risk of Bias Assessment

Outcome-specific RoB 2 judgments were summarized for the 13 included independent randomized controlled trials (Figure 2). No assessed result was judged at high overall risk of bias, whereas the displayed result for all 13 trials was rated as having some concerns overall. By domain, D1 (randomization process) was rated low risk in 3 trials and some concerns in 10; D2 (deviations from intended interventions) was rated low risk in 10 trials and some concerns in 3; D3 (missing outcome data) was rated low risk in 5 trials and some concerns in 8; D4 (measurement of the outcome) was rated low risk in all 13 trials; and D5 (selection of the reported result) was rated as some concerns in all 13 trials. The predominant source of concern was therefore selection of the reported result, with additional uncertainty related to randomization and missing outcome data in several trials. The excluded Shafabakhsh et al. [25] (T2DM + CHD) report was not included in the RoB 2 assessment. Supplementary Table S6 specifies the representative inflammatory result for each trial and provides the result-level rationale for each domain judgment.

### 3.4. Quantitative Synthesis

#### 3.4.1. Effect of Curcumin Supplementation on hs-CRP

**Figure 3:**
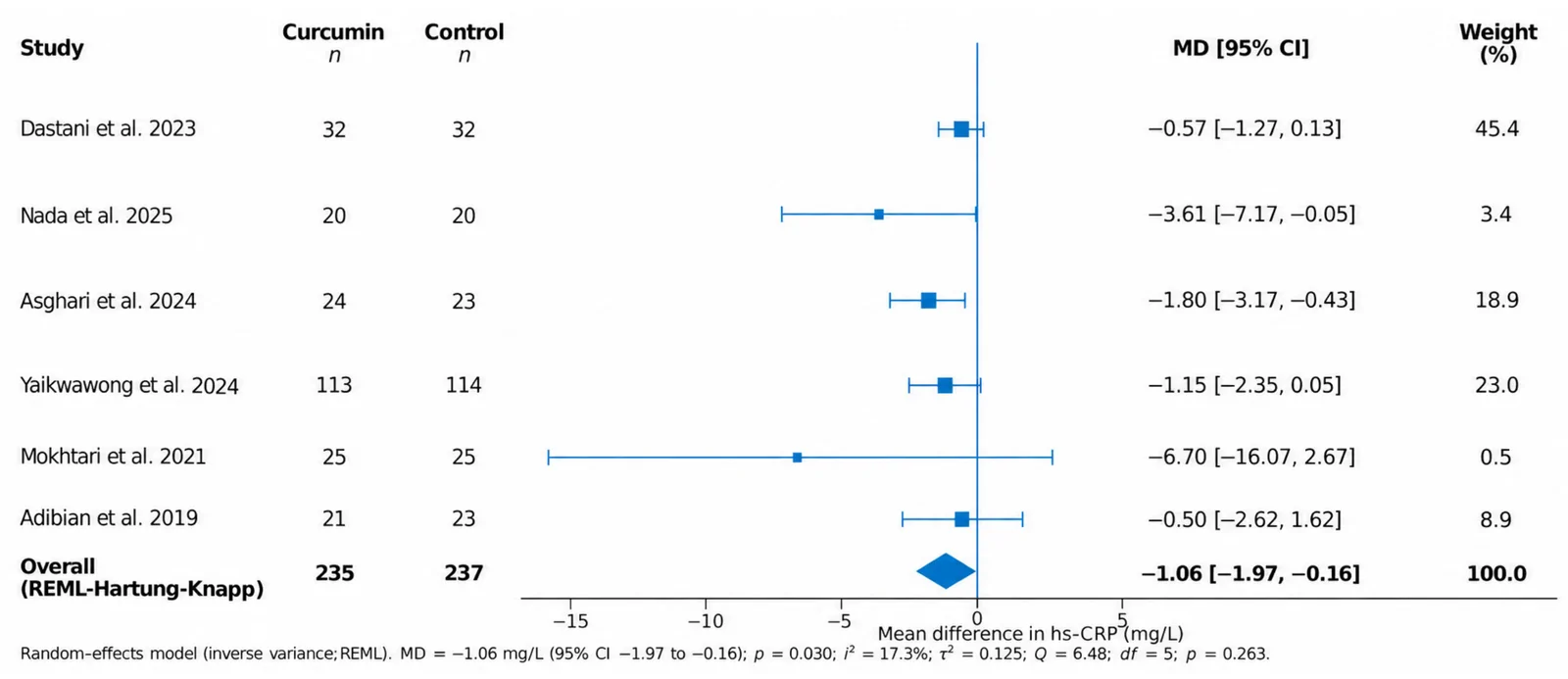
Forest plot of the effect of oral curcumin/curcuminoid supplementation on circulating high-sensitivity C-reactive protein (hs-CRP) concentrations in adults with type 2 diabetes mellitus (T2DM). Six independent randomized controlled trials comprising 472 participants were included in the primary endpoint mean-difference synthesis. Estimates were pooled using an inverse-variance random-effects model with restricted maximum likelihood (REML) estimation and Hartung–Knapp-adjusted 95% confidence intervals. The pooled estimate favored curcumin (MD = −1.06 mg/L; 95% CI −1.97 to −0.16; *p* = 0.030), with low heterogeneity (*I*^2^ = 17.3%; τ^2^ = 0.125; *Q* = 6.48, *df* = 5, *p* = 0.263). Negative mean differences favor curcumin. Studies shown, from top to bottom: Dastani et al. [35], Nada et al. [34], Asghari et al. [32], Yaikwawong et al. [31], Mokhtari et al. [42], and Adibian et al. [40].

Six independent randomized controlled trials comprising 472 participants contributed compatible endpoint data to the primary hs-CRP meta-analysis. Using an inverse-variance random-effects model with REML estimation and Hartung–Knapp-adjusted 95% confidence intervals, oral curcumin/curcuminoid supplementation was associated with lower circulating hs-CRP compared with control (MD = −1.06 mg/L; 95% CI −1.97 to −0.16; *p* = 0.030). Between-study heterogeneity was low (*I*^2^ = 17.3%; τ^2^ = 0.125; *Q* = 6.48, *df* = 5, *p* = 0.263) (Figure 3).

#### 3.4.2. Sensitivity Analysis

A Wald-type sensitivity analysis yielded the same pooled point estimate with a narrower interval (MD = −1.06 mg/L; 95% CI −1.73 to −0.40; *p* = 0.002). Restricting the synthesis to the four trials reporting endpoint means and standard deviations directly (Dastani et al. [35], Asghari et al. [32], Mokhtari et al. [42], and Adibian et al. [40]; *n* = 205) preserved the direction of effect but increased imprecision (MD = −0.95 mg/L; 95% CI −2.36 to 0.45; *p* = 0.120; *I*^2^ = 24.0%).

Panahi et al. [28] was evaluated in a unit-invariant standardized-mean-difference sensitivity analysis because the publication reported an implausible hs-CRP unit. When this trial was added to the six primary hs-CRP trials, the pooled standardized effect remained directionally favorable but crossed the null (Hedges’ g = −0.28; 95% CI −0.58 to 0.02; *p* = 0.062; *I*^2^ = 46.1%). This analysis indicates that the hs-CRP inference is partly sensitive to the handling of unit-ambiguous data.

The hemodialysis trial by Shafabakhsh et al. [29] reported a baseline-adjusted hs-CRP effect (beta = −0.78 mg/L; 95% CI −1.41 to −0.15) but did not provide directly verified group-level endpoint means and standard deviations suitable for the primary mean-difference pool. A separate generic inverse-variance sensitivity analysis combining this adjusted estimate with the six unadjusted endpoint contrasts yielded an exploratory pooled estimate of approximately −0.88 mg/L (95% CI −1.41 to −0.35; *p* = 0.007; *I*^2^ = 0%). Because this analysis combines adjusted and unadjusted estimands, it should not replace the primary analysis. Mansour et al. [27] was likewise retained as an exploratory sensitivity analysis because its baseline-adjusted ANCOVA could not be reconstructed from the reported summary statistics.

Leave-one-out analyses did not reverse the direction of the primary hs-CRP estimate. Pooled mean differences ranged from approximately −0.80 to −1.45 mg/L and *I*^2^ from 0% to 31.3%. Excluding Adibian et al. [40] returned the previous five-trial estimate and caused the Hartung–Knapp interval to marginally include the null (MD = −1.19 mg/L; 95% CI −2.40 to 0.01; *p* = 0.051; *I*^2^ = 30.6%). Overall, the primary inception-based analysis favored curcumin, but the precision of the conclusion remained sensitive to individual studies and to the handling of nonstandard data reporting (Table 3).

#### 3.4.3. Effect of Curcumin Supplementation on TNF-α

**Figure 4:**
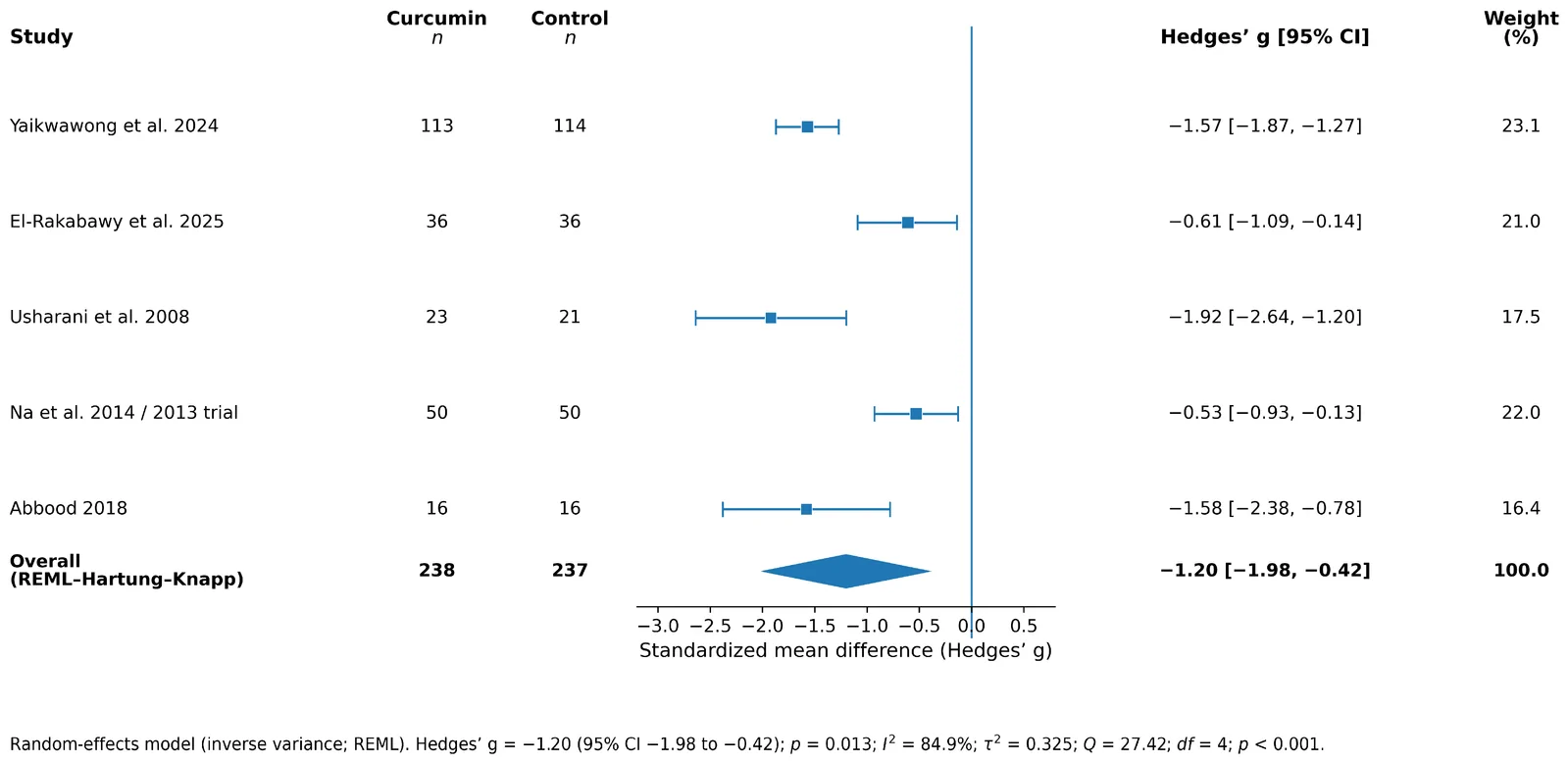
Forest plot of the effect of oral curcumin/curcuminoid supplementation on circulating TNF-α concentrations in adults with T2DM. Five independent randomized controlled trials with usable group-level data (*n* = 475) were quantitatively synthesized using Hedges’ g and an inverse-variance random-effects model with REML estimation and Hartung–Knapp-adjusted 95% confidence intervals. The pooled effect favored curcumin (Hedges’ g =−1.20; 95% CI−1.98 to −0.42; *p* = 0.013), with substantial heterogeneity (*I*^2^ = 84.9%; τ^2^ = 0.325; *Q* = 27.42, *df* = 4, *p* < 0.001). Negative standardized mean differences favor curcumin. Studies shown, from top to bottom: Yaikwawong et al. [31], El-Rakabawy et al. [33], Usharani et al. [36], Na et al. [37,38], and Abbood [41].

Five independent randomized controlled trials comprising 475 participants provided usable group-level TNF-α data. Because outcome distributions and scales differed materially across studies, standardized mean differences with Hedges’ small-sample correction were pooled. The REML–Hartung–Knapp random-effects model favored curcumin supplementation (Hedges’ g = −1.20; 95% CI −1.98 to −0.42; *p* = 0.013), although substantial between-study heterogeneity was present (*I*^2^ = 84.9%; τ^2^ = 0.325; *Q* = 27.42, *df* = 4, *p* < 0.001) (Figure 4).

A sixth eligible randomized trial from the Panahi/IRCT201505301165N4 cohort measured TNF-α and reported a significant between-group reduction, but the accessible report did not provide the group-level TNF-α values required for an auditable standardized effect. It was therefore retained narratively rather than counted as an additional quantitative study. Leave-one-out analyses preserved the favorable direction of effect, but substantial heterogeneity remained, underscoring uncertainty regarding the magnitude of the pooled effect.

### 3.5. Narrative Synthesis of IL-6 and IL-1β

The expanded inception search identified IL-6 evidence from three independent randomized cohorts. In Yaikwawong et al. [31], IL-6 at 12 months was lower with curcumin than with placebo (6.12 vs. 15.84 pg/mL), and Usharani et al. [36] reported lower post-treatment IL-6 with NCB-02 than with placebo (1.83 ± 0.67 vs. 3.57 ± 2.21 pg/mL). Na et al. [38], a secondary inflammatory-biomarker report from the 2013 ISRCTN85826075 trial, reported follow-up IL-6 values of 2.68 ± 1.63 pg/mL in the intervention group and 3.90 ± 2.04 pg/mL in the control group. Between-group follow-up inference in the Na report used ANCOVA adjusted for baseline values and covariates (Supplementary Table S5, Panel C).

Although group-level numerical IL-6 data were reported for all three cohorts, no pooled IL-6 estimate was included in the final analysis. IL-6 was retained as a narrative outcome because the evidence base was small and there were substantial differences in intervention duration, formulation, outcome distribution/reporting, and analytical approach, including the ANCOVA-adjusted inference in the Na secondary report. The designation “narrative” describes the final synthesis role, not the absence of numerical outcome data. IL-1β remained available from one randomized cohort (Yaikwawong et al. [31]), which showed a favorable reduction over 12 months. These findings suggest a possible broader anti-inflammatory signal but do not permit precise quantitative conclusions for IL-6 or IL-1β.

### 3.6. Certainty of Evidence (GRADE)

The certainty of evidence was assessed using GRADE after completion of the expanded search and RoB 2 evaluation (Table 4). For hs-CRP, certainty was rated as moderate. The evidence was downgraded one level for risk of bias because the contributing trials were judged as having some concerns overall, driven primarily by selection of the reported result and, in several trials, additional concerns related to randomization and missing outcome data. Inconsistency was not considered serious because heterogeneity was low (*I*^2^ = 17.3%); indirectness and imprecision were not rated as serious for the primary estimate. Although the sensitivity analysis restricted to four trials with directly reported endpoint means and standard deviations crossed the null, its effect direction remained favorable; this loss of precision was treated as a robustness limitation rather than as an additional GRADE downgrade. Publication bias was not downgraded because no specific evidence of publication bias was identified; however, formal assessment was not feasible with fewer than ten trials, and this limitation warrants caution. The sensitivity of the hs-CRP inference to unit handling and data preparation was considered in the interpretation of the final rating.

For TNF-α, certainty was rated as low. The evidence was downgraded one level for risk of bias because the contributing trials were judged as having some concerns overall and one level for serious inconsistency because between-study heterogeneity was substantial (*I*^2^ = 84.9%). Indirectness and imprecision were not rated as serious for the primary estimate, whose confidence interval excluded the null, although the magnitude of effect remained variable. Publication bias was not downgraded because no specific evidence of publication bias was identified; however, fewer than ten trials contributed to the synthesis, precluding informative formal asymmetry testing. One additional eligible TNF-α trial could not be incorporated quantitatively because extractable group-level data were unavailable, further supporting cautious interpretation of the effect magnitude.

The final GRADE ratings were therefore moderate certainty for hs-CRP and low certainty for TNF-α. These judgments support a favorable direction of effect for both biomarkers while acknowledging limitations related to risk of bias, sensitivity of the hs-CRP analysis to data-handling decisions, and substantial inconsistency in the TNF-α evidence base.

## 4. Discussion

### 4.1. Principal Findings

This systematic review and meta-analysis evaluated oral curcumin/curcuminoid supplementation and inflammatory biomarkers in adults with T2DM using an expanded inception search. The primary hs-CRP synthesis of six RCTs (*n* = 472) favored curcumin (MD = −1.06 mg/L; 95% CI −1.97 to −0.16) with low heterogeneity, whereas the TNF-α synthesis of five RCTs (*n* = 475) also favored curcumin (Hedges’ g = −1.20; 95% CI −1.98 to −0.42) but showed substantial heterogeneity. IL-6 evidence from three randomized cohorts was directionally favorable but too clinically and methodologically heterogeneous to support a stable pooled estimate, and IL-1β remained supported by a single randomized cohort.

An important methodological finding was that the inference varied according to how nonstandard outcome reporting was handled. For hs-CRP, the primary endpoint mean-difference pool was statistically significant, whereas a unit-invariant sensitivity analysis incorporating Panahi et al. [28] crossed the null. Conversely, a sensitivity analysis incorporating the baseline-adjusted hs-CRP effect from the hemodialysis trial remained favorable, although it combined adjusted and unadjusted estimands. These analyses underscore the importance of transparent trial-level extraction, explicit handling of converted data, and separation of incompatible estimands rather than presenting a single pooled estimate as unequivocal.

Taken together, the expanded evidence supports a potential anti-inflammatory effect of curcumin in T2DM, particularly for hs-CRP and TNF-α. Nevertheless, these results should not be interpreted as evidence of a uniform class effect across all curcumin formulations or clinical subgroups. Differences in formulation, bioavailability, dose, intervention duration, background therapy, and comorbidity may plausibly contribute to the observed variability and remain important priorities for future research.

### 4.2. Effect of Curcumin Supplementation on hs-CRP

The inception-based hs-CRP analysis showed a pooled reduction of approximately 1.06 mg/L with curcumin/curcuminoid supplementation, with low statistical heterogeneity (*I*^2^ = 17.3%). This result differs from the earlier restricted analysis because Adibian et al. [40] was recovered by the expanded search and contributed compatible endpoint data. Importantly, the favorable primary estimate was sensitive to some analytic choices: excluding Adibian returned the previous borderline Hartung–Knapp result, and adding Panahi et al. [28] through a unit-invariant SMD sensitivity analysis also produced a confidence interval that included the null.

Previous evidence syntheses provide a useful benchmark for the present estimates but do not support a uniform conclusion. In 18 trials of adults with T2DM, Mokgalaboni et al. [21] reported lower CRP with curcumin (SMD = −0.59; 95% CI −1.11 to −0.07), accompanied by substantial heterogeneity (*I*^2^ = 77%). By contrast, Liang [20] pooled 11 RCTs (645 participants) and found that the hs-CRP effect did not reach statistical significance (SMD = −0.35; 95% CI −0.73 to 0.03; *I*^2^ = 73%). Bahari et al. [22] observed significant reductions in CRP, TNF-α, and IL-6, but that review combined prediabetes with T2DM, considered inflammatory and oxidative-stress outcomes together, and judged the overall certainty to be low. Umbrella reviews have likewise suggested an anti-inflammatory signal across multiple clinical populations, although their broad populations limit direct applicability to adults with established T2DM.

The present analysis therefore helps explain some of the inconsistency across earlier syntheses. Differences in eligible publication windows, treatment formulations, effect measures, unit verification, and statistical reconstruction materially influence which trials contribute to the quantitative pool. The primary estimate includes only endpoint contrasts that could be expressed comparably in mg/L, whereas unit-ambiguous and adjusted-only effects were retained in explicit sensitivity analyses. This approach provides a clinically interpretable estimate while preserving transparency regarding residual uncertainty arising from nonuniform reporting.

The favorable hs-CRP signal is biologically plausible. High-sensitivity C-reactive protein is an acute-phase reactant synthesized predominantly in response to inflammatory cytokine signaling, particularly IL-6. Curcumin can inhibit NF-κB signaling, attenuate oxidative stress, and reduce transcription of pro-inflammatory mediators. These mechanisms support the observed direction of effect, but mechanistic plausibility should not override uncertainty arising from trial-level reporting and sensitivity analyses.

From a clinical perspective, hs-CRP is associated with systemic inflammation and cardiovascular risk in T2DM. However, none of the pooled analyses establishes that an hs-CRP reduction of this magnitude translates into fewer cardiovascular events, improved glycemic outcomes, or better long-term prognosis. Future trials should prespecify inflammatory outcomes, report both raw group-level data and adjusted treatment effects, and evaluate whether biomarker changes are accompanied by clinically meaningful outcomes.

### 4.3. Effect of Curcumin Supplementation on TNF-α

The expanded TNF-α synthesis provides a broader quantitative evidence base than the previous two-trial analysis. Five randomized controlled trials (RCTs) involving 475 participants contributed group-level data, and the pooled standardized effect favored curcumin (Hedges’ g = −1.20; 95% CI −1.98 to −0.42). Substantial heterogeneity was observed (*I*^2^ = 84.9%), indicating considerable variation in effect magnitude across formulations, populations, and study designs. An additional eligible Panahi trial reported a significant reduction in TNF-α but could not be included in the quantitative synthesis because the accessible report did not provide sufficient group-level data.

TNF-α is a central pro-inflammatory cytokine implicated in the pathogenesis of T2DM. It contributes directly to insulin resistance by promoting serine phosphorylation of insulin receptor substrate proteins, thereby impairing insulin signaling and glucose uptake in peripheral tissues [3,5]. TNF-α also activates the NF-κB signaling pathway, sustaining chronic low-grade inflammation and stimulating the production of additional pro-inflammatory mediators. Persistent TNF-α elevation is associated with endothelial dysfunction, oxidative stress, pancreatic β-cell dysfunction, and increased cardiovascular risk in individuals with T2DM [6,8].

The TNF-α findings are also consistent with experimentally described actions of curcumin. Studies of inflammatory signaling indicate that curcumin can limit NF-κB activation, attenuate macrophage activity and transcription of TNF-α, IL-6, and IL-1β, and enhance antioxidant responses through Nrf2 activation [12,14,16]. These convergent effects on cytokine signaling and oxidative stress provide a plausible mechanistic context for the lower circulating TNF-α observed in the quantitative synthesis without establishing mechanism as the cause of the clinical effect.

Accordingly, the TNF-α findings should be interpreted as evidence of a favorable average effect rather than a precise estimate of a uniform treatment response. The final GRADE certainty for TNF-α was low because of risk-of-bias concerns and substantial inconsistency across trials. Further randomized trials using standardized TNF-α assays and comprehensive group-level outcome reporting are needed to clarify which formulations and patient populations are most likely to benefit.

### 4.4. Biological Mechanisms Underlying the Anti-Inflammatory Effects of Curcumin

**Figure 5:**
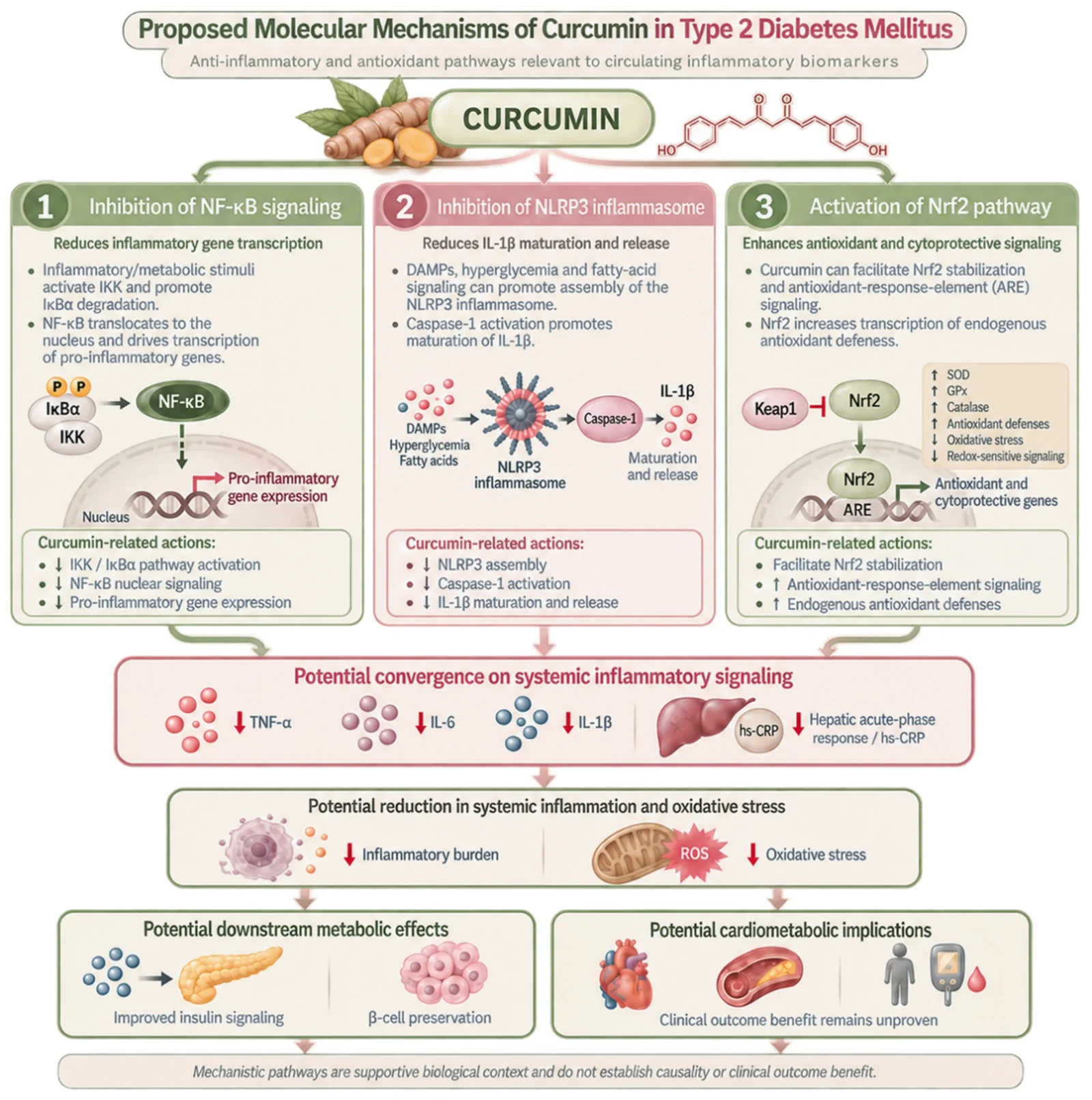
Proposed molecular mechanisms through which curcumin may influence inflammation in adults with type 2 diabetes mellitus. Curcumin can inhibit NF-κB signaling and the NLRP3 inflammasome while activating Nrf2-related antioxidant pathways, potentially reducing pro-inflammatory cytokine production, oxidative stress, and the hepatic acute-phase response. Downstream metabolic and cardiometabolic effects are presented as mechanistic hypotheses rather than demonstrated clinical outcomes.

The favorable biomarker findings identified in this systematic review are biologically plausible in light of established molecular pathways through which curcumin can modulate inflammatory signaling (Figure 5). Although hs-CRP and TNF-α were the only biomarkers included in quantitative syntheses, evidence for IL-6 and IL-1β, together with extensive experimental data, suggests that curcumin may influence several interconnected inflammatory pathways [12,14].

One principal mechanism involves inhibition of the nuclear factor kappa B (NF-κB) signaling pathway. NF-κB functions as a major regulator of inflammatory gene transcription and is central to the chronic low-grade inflammatory state characteristic of type 2 diabetes mellitus (T2DM) [3,5]. Persistent NF-κB activation promotes expression of pro-inflammatory cytokines, including TNF-α, IL-6, and IL-1β, which contribute to insulin resistance, endothelial dysfunction, oxidative stress, and progressive pancreatic β-cell dysfunction [3,6]. Experimental studies consistently show that curcumin can suppress NF-κB activation and thereby reduce transcription of these inflammatory mediators [14,16].

Curcumin has also been shown to inhibit activation of the NLRP3 inflammasome, thereby decreasing caspase-1 activation and the subsequent maturation and release of IL-1β [14,16]. This mechanism may contribute to preservation of pancreatic β-cell function and attenuation of inflammatory processes associated with insulin resistance and metabolic dysfunction. Although quantitative clinical evidence for IL-1β remains limited, the available findings are consistent with this proposed mechanism.

In addition to its direct anti-inflammatory actions, curcumin can activate the nuclear factor erythroid 2-related factor 2 (Nrf2) signaling pathway. Nrf2 activation increases expression of endogenous antioxidant enzymes, including superoxide dismutase, glutathione peroxidase, and catalase, thereby reducing oxidative stress [12,16]. Because oxidative stress and inflammation can reinforce one another through NF-κB activation, attenuation of oxidative damage may indirectly contribute to the observed reductions in circulating hs-CRP and TNF-α concentrations.

Collectively, these mechanisms provide a coherent biological rationale for the favorable biomarker findings. The observed reductions in hs-CRP and TNF-α may reflect coordinated modulation of inflammatory and oxidative-stress pathways rather than isolated effects on individual biomarkers. However, the substantial heterogeneity observed for TNF-α and the sensitivity of hs-CRP to data-handling decisions indicate that mechanistic plausibility should be considered supportive context rather than definitive evidence of clinical efficacy.

### 4.5. Narrative Synthesis of IL-6 and IL-1β

The inception search identified IL-6 evidence from three independent randomized cohorts. Usharani et al. [36] also observed lower post-treatment IL-6 following standardized curcuminoid administration, whereas Na et al. [38] reported a significant adjusted reduction in IL-6 in a secondary analysis of the 2013 randomized trial. Collectively, these findings suggest a consistent association between curcumin or curcuminoid supplementation and lower IL-6 concentrations. However, the included studies differed substantially in intervention duration, formulation, outcome measurement, and statistical reporting.

Although all three cohorts reported numerical group-level IL-6 values, IL-6 was retained as a narrative outcome in the final analysis because the small evidence base was clinically and methodologically heterogeneous. Differences concerned intervention duration, formulation, outcome distribution/reporting, and analytical approach; in particular, the Na secondary report used ANCOVA-adjusted between-group follow-up inference. The decision not to pool IL-6 therefore reflects limited comparability across the three cohorts, not an absence of a reported control-group endpoint (Supplementary Table S5, Panel C). Evidence for IL-1β remains limited to the Yaikwawong randomized cohort, which showed a favorable 12-month trend.

Both IL-6 and IL-1β are biologically relevant to T2DM. IL-6 promotes hepatic acute-phase protein synthesis, including CRP, and contributes to insulin resistance and endothelial dysfunction. IL-1β, a downstream product of NLRP3 inflammasome activation, contributes to β-cell dysfunction and metabolic inflammation. Inhibition of NF-κB and NLRP3 signaling by curcumin provides a plausible mechanistic explanation for the favorable cytokine profiles observed in these studies.

Nevertheless, the narrative cytokine evidence should be considered secondary to the more rigorously defined quantitative analyses of hs-CRP and TNF-α. Standardized assay methods, comprehensive reporting of group-level data, and prespecified cytokine outcomes are needed before IL-6 or IL-1β can support precise pooled estimates or clinical recommendations.

In summary, the expanded evidence base suggests that curcumin may modulate multiple interconnected inflammatory mediators. Although the direction of effects across biomarkers is generally favorable, substantial heterogeneity and incomplete reporting preclude a definitive conclusion regarding a broad anti-inflammatory class effect in T2DM (Table 5).

### 4.6. Strengths and Limitations

This review has several strengths. The final search update extended to database inception and used a sensitivity-first strategy without a mandatory biomarker block, reducing the likelihood of omitting randomized trials in which inflammatory outcomes were secondary. Overlapping publications were systematically mapped to independent trial cohorts. Analyses of hs-CRP and TNF-α used effect measures appropriate to their respective data structures, and nonstandard or adjusted-only data were assigned to explicit sensitivity analyses rather than combined with the primary estimands. The use of RoB 2, GRADE, and leave-one-out analyses further strengthened methodological transparency.

Several limitations should also be considered. The number of trials contributing to each quantitative biomarker remained limited, and substantial clinical variability was present in formulations, doses, intervention durations, comorbidities, background therapies, and biomarker measurement methods. TNF-α showed substantial heterogeneity. For hs-CRP, some trials required statistical preparation of dispersion data; Panahi et al. [28] reported an implausible printed unit, and adjusted-only effects from the hemodialysis and Mansour trials were not directly comparable with unadjusted endpoint contrasts. IL-6 reporting remained insufficiently homogeneous to support a stable pooled estimate. Publication bias could not be robustly assessed because fewer than ten independent trials contributed to each synthesis.

The final electronic search was conducted in PubMed, Scopus, and the Web of Science Core Collection. CENTRAL and Ovid MEDLINE could not be included in the final update because complete institutional access and/or export functionality was unavailable. Consequently, eligible reports indexed exclusively in these sources may have been missed. This limitation was partially mitigated by the broad inception-based search strategy, which did not require an inflammatory-biomarker term, together with citation searching of eligible trials and relevant reviews. Nevertheless, residual retrieval bias cannot be excluded. The completed RoB 2 assessment identified some concerns overall across the 13 included trials and should be interpreted alongside the quantitative findings and final GRADE ratings.

### 4.7. Clinical Implications and Future Perspectives

Current evidence suggests that curcumin or curcuminoid supplementation may have adjunctive potential for modulating systemic inflammation in adults with T2DM. Although the primary hs-CRP and TNF-α analyses favored curcumin, the hs-CRP findings were sensitive to nonstandard data handling and the TNF-α effects were highly variable across trials. These biomarker findings do not support replacement of established pharmacological, nutritional, or lifestyle management and do not justify routine curcumin use for prevention of clinical complications.

Future RCTs should use standardized, chemically characterized formulations, prespecify inflammatory biomarkers, harmonize laboratory methods and reporting units, and provide both raw group-level endpoint data and adjusted treatment effects. Larger sample sizes and longer follow-up are needed to determine whether reductions in inflammatory biomarkers translate into meaningful improvements in glycemic control, cardiovascular events, diabetic complications, or quality of life.

## 5. Conclusions

This expanded systematic review and meta-analysis identified a favorable average effect of oral curcumin or curcuminoid supplementation on hs-CRP and TNF-α concentrations in adults with type 2 diabetes mellitus (T2DM). Six randomized controlled trials (RCTs; *n* = 472) contributed to the primary hs-CRP synthesis (MD = −1.06 mg/L; 95% CI −1.97 to −0.16; *I*^2^ = 17.3%), whereas five RCTs (*n* = 475) provided quantitative TNF-α data (Hedges’ g = −1.20; 95% CI −1.98 to −0.42; *I*^2^ = 84.9%).

The hs-CRP result was sensitive to the handling of unit-ambiguous and adjusted-only data, whereas the TNF-α estimate showed substantial inconsistency in effect magnitude. Evidence for IL-6 from three randomized cohorts was directionally favorable but remained too clinically and methodologically heterogeneous to support a stable pooled estimate. IL-1β findings were limited to a single randomized cohort. Collectively, these findings are biologically plausible but do not establish a uniform or definitive anti-inflammatory effect across all curcumin formulations and T2DM populations.

The certainty of evidence was moderate for hs-CRP and low for TNF-α. These final GRADE ratings incorporate the completed RoB 2 assessment, the low heterogeneity of the primary hs-CRP synthesis, and the substantial inconsistency observed for TNF-α. Curcumin should therefore be considered a potentially useful adjunctive intervention that requires further confirmation in well-designed, adequately powered randomized trials before routine clinical recommendations can be established.

## Figures and Tables

**Table 1:** Eligibility criteria according to the PICOS framework.

PICOS Component	Eligibility Criteria
Population	Adults (≥18 years) with type 2 diabetes mellitus
Intervention	Oral curcumin supplementation (all formulations and doses)
Comparator	Placebo or standard care
Outcomes	hs-CRP, TNF-α, IL-6, IL-1β
Study design	Randomized controlled trials

**Table 3:** Sensitivity analyses for the pooled effect of curcumin/curcuminoid supplementation on hs-CRP. MD, mean difference; SMD, standardized mean difference; CI, confidence interval; REML, restricted maximum likelihood; SD, standard deviation. Negative effects favor curcumin. Adjusted-effect and Mansour exploratory analyses are described in the text because their estimands are not directly equivalent to the primary endpoint MD.

Analysis	Studies	Participants	Effect (95% CI)	*p*-Value	*I* ^2^	Interpretation
Primary REML–Hartung–Knapp MD	6	472	−1.06 mg/L (−1.97 to −0.16)	0.030	17.3%	Favors curcumin; statistically significant in the primary inception analysis
REML–Wald sensitivity	6	472	−1.06 mg/L (−1.73 to −0.40)	0.002	17.3%	Same direction and inference
Direct endpoint mean/SD only	4	205	−0.95 mg/L (−2.36 to 0.45)	0.120	24.0%	Direction unchanged; greater imprecision
Unit-invariant SMD including Panahi 2018 [28]	7	572	Hedges’ g −0.28 (−0.58 to 0.02)	0.062	46.1%	Direction favorable; CI includes the null

**Table 4:** Summary of Findings (GRADE) for the primary quantitative inflammatory outcomes. RCT, randomized controlled trial; MD, mean difference; CI, confidence interval; GRADE, Grading of Recommendations Assessment, Development and Evaluation. RCT evidence started at high certainty and was downgraded according to the domains shown in the table. GRADE certainty ratings: ⊕⊕⊕⊕, high; ⊕⊕⊕○, moderate; ⊕⊕○○, low; and ⊕○○○, very low certainty of evidence. Filled circles (⊕) indicate retained levels of certainty, and empty circles (○) indicate levels not attained after downgrading.

Outcome	Quantitative RCTs	Participants	Primary Effect	GRADE Assessment and Downgrade Rationale	Certainty of Evidence
hs-CRP	6	472	MD −1.06 mg/L (95% CI −1.97 to −0.16)	Risk of bias: serious (−1). Inconsistency: not serious (*I*^2^ = 17.3%). Indirectness: not serious. Imprecision: not serious for the primary estimate. Publication bias: not downgraded; no specific evidence identified, but formal assessment not feasible (<10 trials).	Moderate (⊕⊕⊕○)
TNF-α	5 (+1 narrative)	475	Hedges’ g −1.20 (95% CI −1.98 to −0.42)	Risk of bias: serious (−1). Inconsistency: serious (−1; *I*^2^ = 84.9%). Indirectness: not serious. Imprecision: not serious for the primary estimate. Publication bias: not downgraded; no specific evidence identified, but formal assessment not feasible (<10 trials).	Low (⊕⊕○○)

**Table 5:** Integration of inflammatory biomarkers evaluated in the expanded systematic review. Quantitative significance should be interpreted together with heterogeneity, sensitivity analyses, and the final GRADE assessment.

Biomarker	Evidence Synthesis	Direction of Effect	Interpretive Note/Biological Context
**hs-CRP**	Meta-analysis: 6 RCTs, *n* = 472	Favors curcumin; significant in primary MD	Low heterogeneity, but sensitivity to unit/data-preparation decisions
**TNF-α**	Meta-analysis: 5 RCTs, *n* = 475 (+1 narrative RCT)	Favors curcumin; significant average effect	Substantial heterogeneity; NF-κB-related mechanisms are biologically plausible
**IL-6**	Narrative synthesis from 3 RCTs	Generally favorable	Small evidence base and substantial clinical/methodological heterogeneity; numerical group-level data reported, but no pooled estimate.
**IL-1β**	Narrative synthesis from 1 randomized cohort	Favorable single-trial signal	NLRP3 inflammasome inhibition provides mechanistic plausibility

## Data Availability

No new data were created or analyzed in this study. Data sharing is not applicable to this article.

## Supplementary Materials

The following supporting information can be downloaded at https://www.mdpi.com/article/10.3390/nu18183028/s1, Table S1: Reconstructed database-specific search strategies and coverage of the final electronic search; Table S2: Full-text assessment and final report-level disposition; Table S3: Included reports mapped to independent randomized trial families; Table S4: Characteristics of the 13 independent randomized controlled trials; Table S5: Outcome-level extraction, data preparation, and analytical role; Table S6: Detailed result-specific Cochrane RoB 2 assessment; Table S7: GRADE evidence profile for the primary quantitative outcomes; Table S8: Sensitivity, subgroup, and leave-one-out analyses.

## Appendix A

**Table A1:** Database search strategies, coverage dates, and numbers of records retrieved before deduplication.

Database	Search Syntax/Coverage	Records Retrieved (*n*)
PubMed	Inception to 31 Aug 2026. Search syntax: (“Curcumin”[Mesh] OR curcumin[Title/Abstract] OR curcuminoid*[Title/Abstract] OR turmeric[Title/Abstract] OR “Curcuma longa”[Title/Abstract]) AND (“Diabetes Mellitus, Type 2”[Mesh] OR “type 2 diabetes mellitus”[Title/Abstract] OR “type 2 diabetes”[Title/Abstract] OR “type II diabetes”[Title/Abstract] OR T2DM[Title/Abstract])	219
Scopus	Inception to 31 Aug 2026. Search syntax: TITLE-ABS-KEY(curcumin OR curcuminoid* OR turmeric OR “Curcuma longa”) AND TITLE-ABS-KEY(“type 2 diabetes mellitus” OR “type 2 diabetes” OR “type II diabetes” OR T2DM)	465
Web of Science Core Collection	Inception to 31 Aug 2026. Search syntax: TS = (curcumin OR curcuminoid* OR turmeric OR “Curcuma longa”) AND TS = (“type 2 diabetes mellitus” OR “type 2 diabetes” OR “type II diabetes” OR T2DM)	577
TOTAL	Database records used in deduplication	1261

The final expanded electronic searches were completed on 31 August 2026. PubMed, Scopus, and Web of Science were included in the deduplication and screening dataset (1261 database records). CENTRAL and Ovid MEDLINE were not included in the final search update because complete access and/or export functionality was unavailable and therefore are not represented in the PRISMA counts. The database-specific strategies reported above document the final searchable sources used for the review. **Note:** *n*, number of records retrieved. The asterisk (*) is a wildcard or truncation symbol used to retrieve all terms beginning with the preceding word stem; for example, curcuminoid* retrieves both “curcuminoid” and “curcuminoids”. TS, Topic Search; TITLE-ABS-KEY, title, abstract, and keywords.
