# Supplementary material for: Effects of Curcumin Supplementation on Inflammatory Biomarkers in Adults with Type 2 Diabetes Mellitus: A Systematic Review and Meta-Analysis of Randomized Controlled Trials
Source: Nutrients. 2026 Sep 16;18(18):3028. doi: 10.3390/nu18183028 (PMC13610151; doi:10.3390/nu18183028)
Supplement: Supplementary file 1 [file nutrients-18-03028-s001.zip › nutrients-4527730-supplementary.pdf]

Supplementary Information

Effects of Curcumin Supplementation on Inflammatory Biomarkers in Adults with Type 2 Diabetes Mellitus: A Systematic Review and Meta-Analysis of Randomized Controlled Trials

Claudio Ignacio Pérez; Jorge Enrique González-Casanova; Diana Marcela Rojas-Gómez

Tables S1–S8

Source provenance and final reconciliation. Tables S2–S8 were assembled from the dual-review screening closeout, PRISMA closeout, audited trial/outcome extraction workbooks, outcome-specific meta-analysis workbooks, and RoB 2 consensus workbook closed on 31 August–1 September 2026. Final eligibility was reconciled to the submitted manuscript: the Shafabakhsh et al. 2020 T2DM + coronary heart disease report (R0837/T14) was excluded at full-text because it did not report a prespecified eligible circulating biomarker outcome. Accordingly, the final evidence base contains 23 reports representing 13 independent randomized trials. Where the locked database audit preserved the search concept and counts but not the literal platform history, Table S1 labels the database syntax as a reproducible reconstruction rather than an exact exported query. The final package was reconciled to the submission-ready manuscript dated 6 September 2026.

Abbreviations: CHD, coronary heart disease; GRADE, Grading of Recommendations Assessment, Development and Evaluation; HD, hemodialysis; hs-CRP, high-sensitivity C-reactive protein; IL, interleukin; MD, mean difference; RCT, randomized controlled trial; REML, restricted maximum likelihood; RoB 2, Cochrane Risk of Bias 2; SMD, standardized mean difference; TNF-α, tumor necrosis factor-α.

Table S1. Reconstructed database-specific search strategies and coverage of the final electronic search

Database	Coverage / final date	Reproducible search syntax / search status	Records	Use in final review
PubMed	Inception–31 Aug 2026	("Curcumin"[Mesh] OR curcumin[Title/Abstract] OR curcuminoid*[Title/Abstract] OR turmeric[Title/Abstract] OR "Curcuma longa"[Title/Abstract]) AND ("Diabetes Mellitus, Type 2"[Mesh] OR "type 2 diabetes mellitus"[Title/Abstract] OR "type 2 diabetes"[Title/Abstract] OR "type II diabetes"[Title/Abstract] OR T2DM[Title/Abstract])	219	Included in deduplication and PRISMA
Scopus	Inception–31 Aug 2026	TITLE-ABS-KEY(curcumin OR curcuminoid* OR turmeric OR "Curcuma longa") AND TITLE-ABS-KEY("type 2 diabetes mellitus" OR "type 2 diabetes" OR "type II diabetes" OR T2DM)	465	Included in deduplication and PRISMA
Web of Science Core Collection	Inception–31 Aug 2026	TS=(curcumin OR curcuminoid* OR turmeric OR "Curcuma longa") AND TS=("type 2 diabetes mellitus" OR "type 2 diabetes" OR "type II diabetes" OR T2DM)	577	Included in deduplication and PRISMA
Cochrane CENTRAL	Not included in final update	Not used as a final searchable source because complete institutional access and/or export functionality was unavailable.	—	Not represented in final PRISMA counts
Ovid MEDLINE	Not included in final update	Not used as a final searchable source because complete institutional access and/or export functionality was unavailable.	—	Not represented in final PRISMA counts

The PubMed, Scopus, and Web of Science strategies shown above are reproducible reconstructions of the locked sensitivity-first concept (curcumin/curcuminoids AND T2DM; no mandatory biomarker block). The audit workbook preserved the final search date and database counts but not the literal platform query histories. CENTRAL and Ovid MEDLINE were not included in the final search update because complete institutional access and/or export functionality was unavailable; therefore, neither source is represented in the PRISMA counts. Only English-language full-text articles were eligible, and no geographical restrictions were applied. To mitigate retrieval limitations, the final searchable databases were queried from inception without a mandatory inflammatory-biomarker term, and reference lists of eligible trials and relevant reviews were additionally examined.

Table S2. Full-text assessment and final report-level disposition

Twenty-four reports assessed at full text after the final inception search and other-method identification.

ID	Year	Report	DOI / source	Trial family	Final disposition	Final reason / report role
R0024	2017	Antioxidant effects of curcuminoids in patients with type 2 diabetes mellitus: a randomized controlled trial. [49]	10.1007/s10787-016-0301-4	T09	Include — linked/overlapping report	Linked publication from an included randomized cohort; retained as a report but not counted as an additional independent RCT.
R0025	2025	The effectiveness and safety of nanocurcumin supplementation for diabetic peripheral neuropathy in patients with type 2 diabetes: a randomized double-blind clinical trial. [27]	10.1186/s12937-025-01184-8	T06	Include — anchor/eligible report	Meets final PICOS and contributes eligible inflammatory evidence.
R0042	2026	Curcumin Supplementation Reduces Inflammation, Neutrophil-to-Lymphocyte Ratio (NLR), and Antioxidant Status in Obese Patients with Type 2 Diabetes: A Randomized Controlled Trial. [45]	10.3390/ijms27093854	T01	Include — linked/overlapping report	Linked publication from an included randomized cohort; retained as a report but not counted as an additional independent RCT.
R0044	2024	Curcumin extract improves beta cell functions in obese patients with type 2 diabetes: a randomized controlled trial. [46]	10.1186/s12937-024-01022-3	T01	Include — linked/overlapping report	Linked publication from an included randomized cohort; retained as a report but not counted as an additional independent RCT.
R0072	2024	The effect of curcumin and high-content eicosapentaenoic acid supplementations in type 2 diabetes mellitus patients: a double-blinded randomized clinical trial. [32]	10.1038/s41387-024-00274-6	T02	Include — anchor/eligible report	Meets final PICOS and contributes eligible inflammatory evidence.
R0104	2025	Curcumin Attenuates Liver Steatosis via Antioxidant and Anti-Inflammatory Pathways in Obese Patients with Type 2 Diabetes Mellitus: A Randomized Controlled Trial. [47]	10.3390/ijms26199286	T01	Include — linked/overlapping report	Linked publication from an included randomized cohort; retained as a report but not counted as an additional independent RCT.
R0123	2025	Curcumin for Inflammation Control in Individuals with Type 2 Diabetes Mellitus and Metabolic Dysfunction-Associated Steatotic Liver Disease: A Randomized Controlled Trial. [52]	10.3390/nu17121972	T01	Include — linked/overlapping report	Linked publication from an included randomized cohort; retained as a report but not counted as an additional independent RCT.
R0146	2025	Curcumin supplementation improves the clinical outcomes of patients with diabetes and atherosclerotic cardiovascular risk. [33]	10.1038/s41598-025-09783-5	T03	Include — anchor/eligible report	Meets final PICOS and contributes eligible inflammatory evidence.
R0216	2023	Three months of combination therapy with nano-curcumin reduces the inflammation and lipoprotein (a) in type 2 diabetic patients with mild to moderate coronary artery disease: Evidence of a randomized, double-blinded, placebo-controlled clinical trial. [35]	10.1002/biof.1874	T05	Include — anchor/eligible report	Meets final PICOS and contributes eligible inflammatory evidence.
R0252	2008	Effect of NCB-02, atorvastatin and placebo on endothelial function, oxidative stress and inflammatory markers in patients with type 2 diabetes mellitus - A randomized, parallel-group, placebo-controlled, 8-week study [36]	10.2165/00126839-200809040-00004	T07	Include — anchor/eligible report	Meets final PICOS and contributes eligible inflammatory evidence.
R0293	2025	Effect of adding fenofibrate versus curcumin to glimepiride in patients with type 2 diabetes: a randomized controlled trial. [34]	10.1186/s40360-025-00950-y	T04	Include — anchor/eligible report	Meets final PICOS and contributes eligible inflammatory evidence.
R0296	2024	The Effect of Curcumin on Reducing Atherogenic Risks in Obese Patients with Type 2 Diabetes: A Randomized Controlled Trial. [31]	10.3390/nu16152441	T01	Include — anchor/eligible report	Meets final PICOS and contributes eligible inflammatory evidence.
R0297	2019	The effect of curcumin supplementation on anthropometric indices, insulin resistance and oxidative stress in patients with type 2 diabetes: a randomized, double-blind clinical trial. [51]	10.1186/s13098-019-0437-7	T10	Include — linked/overlapping report	Linked publication from an included randomized cohort; retained as a report but not counted as an additional independent RCT.
R0330	2017	Curcuminoids modify lipid profile in type 2 diabetes mellitus: A randomized controlled trial. [50]	10.1016/j.ctim.2017.05.006	T09	Include — linked/overlapping report	Linked publication from an included randomized cohort; retained as a report but not counted as an additional independent RCT.
R0365	2019	The effects of curcumin supplementation on high-sensitivity C-reactive protein, serum adiponectin, and lipid profile in patients with type 2 diabetes: A randomized, double-blind, placebo-controlled trial. [40]	10.1002/ptr.6328	T10	Include — anchor/eligible report	Meets final PICOS and contributes eligible inflammatory evidence.
R0392	2014	Curcuminoids Target Decreasing Serum Adipocyte-fatty Acid Binding Protein Levels in Their Glucose-lowering Effect in Patients with Type 2 Diabetes. [38]	10.3967/bes2014.127	T08	Include — linked secondary report with original trial data	Secondary inflammatory report from the eligible T08 randomized cohort. Although published in a letter format, it contains original, extractable TNF-α/IL-6 trial data; it was retained, unequivocally linked to the parent RCT, and not counted as an additional independent trial.
R0394	2024	Curcumin Reduces Depression in Obese Patients with Type 2 Diabetes: A Randomized Controlled Trial. [48]	10.3390/nu16152414	T01	Include — linked/overlapping report	Linked publication from an included randomized cohort; retained as a report but not counted as an additional independent RCT.
R0417	2013	Curcuminoids exert glucose-lowering effect in type 2 diabetes by decreasing serum	10.1002/mnfr.201200131	T08	Include — linked/overlapping	Linked publication from an included randomized

Reference numbers correspond to the main manuscript.

ID	Year	Report	DOI / source	Trial family	Final disposition	Final reason / report role
		free fatty acids: a double-blind, placebo-controlled trial. [37]			report	cohort; retained as a report but not counted as an additional independent RCT.
R0596	2018	Effects of Curcuminoids Plus Piperine on Glycemic, Hepatic and Inflammatory Biomarkers in Patients with Type 2 Diabetes Mellitus: A Randomized Double-Blind Placebo-Controlled Trial. [28]	10.1055/s-0044-101752	T09	Include — anchor/eligible report	Meets final PICOS and contributes eligible inflammatory evidence.
R0837	2020	Curcumin administration and the effects on psychological status and markers of inflammation and oxidative damage in patients with type 2 diabetes and coronary heart disease. [25]	10.1016/j.clnesp.2020.09.029	T14	Exclude at full text	No eligible prespecified circulating inflammatory biomarker outcome: inflammatory-related gene expression was reported instead of eligible circulating hs-CRP, TNF-α, IL-6, or IL-1β concentrations.
R0842	2017	Curcuminoids Plus Piperine Modulate Adipokines in Type 2 Diabetes Mellitus. [39]	10.2174/1574884713666180104095641	T09	Include — linked/overlapping report	Linked publication from an included randomized cohort; retained as a report but not counted as an additional independent RCT.
CC01	2018	Abbood [41]	https://globalresearchonline.net/journalcontents/v49-1/01.pdf	T11	Include — other methods	Independent T2DM RCT identified by citation chasing. Use curcumin 1000 mg/day vs placebo; TNF-alpha quantitative. Atorvastatin arm is not part of the curcumin-vs-placebo contrast.
CC02	2021	Mokhtari [42]	https://pubmed.ncbi.nlm.nih.gov/33200488/	T12	Include — other methods	Eligible T2DM + grade 3 diabetic foot ulcer RCT. Population verification resolved as T2DM. Nanocurcumin 80 mg/day for 12 weeks vs placebo; hs-CRP quantitative.
CC03	2020	Shafabakhsh — diabetes on hemodialysis [29]	https://pubmed.ncbi.nlm.nih.gov/32655024/	T13	Include — other methods	Eligible T2DM on maintenance hemodialysis RCT. Population verification resolved as T2DM from linked trial-family/full-text evidence. Nanocurcumin 80 mg/day for 12 weeks vs placebo; adjusted hs-CRP effect available. Quantitative role should remain adjusted-effect/sensitivity unless compatible group-level endpoints are verified.

Final full-text accounting: 24 reports assessed; 1 report excluded (R0837/T14); 23 reports retained. Reports labelled linked/overlapping remain part of the included report set but are mapped to a common trial family and are not counted as additional independent RCTs. Secondary or companion reports, including letters, were retained only when they contained original, extractable data for a prespecified biomarker and could be unequivocally linked to an eligible parent RCT.

Table S3. Included reports mapped to independent randomized trial families

Trial ID	Independent trial / anchor	Included reports (n)	Included report IDs	Report years	Anchor used for outcome extraction	Identification route
T01	Yaikwawong / TCTR20140303003 [31,45,46,47,48,52]	6	R0042, R0044, R0104, R0123, R0296, R0394	2026, 2024, 2025, 2025, 2024, 2024	R0296 (2024 atherogenic-risk full cohort)	Database search
T02	Asghari et al. 2024 [32]	1	R0072	2024	R0072 (2024)	Database search
T03	El-Rakabawy et al. 2025 [33]	1	R0146	2025	R0146 (2025)	Database search
T04	Nada et al. 2025 [34]	1	R0293	2025	R0293 (2025)	Database search
T05	Dastani et al. 2023 [35]	1	R0216	2023	R0216 (2023)	Database search
T06	Mansour et al. 2025 [27]	1	R0025	2025	R0025 (2025)	Database search
T07	Usharani et al. 2008 [36]	1	R0252	2008	R0252 (2008)	Database search
T08	Na et al. 2013/2014 [37,38]	2	R0392, R0417	2014, 2013	R0417 (2013 primary report) + R0392 (2014 secondary inflammatory report with original biomarker data)	Database search
T09	Panahi / IRCT201505301165N4 [28,39,49,50]	4	R0024, R0330, R0596, R0842	2017, 2017, 2018, 2017	R0596 (2018 inflammatory report)	Database search
T10	Adibian / Hodaei cohort [40,51]	2	R0297, R0365	2019, 2019	R0365 (2019 hs-CRP report)	Database search
T11	Abbood et al. 2018 [41]	1	CC01	2018	CC01 (2018; citation chasing)	Other methods
T12	Mokhtari et al. 2021 [42]	1	CC02	2021	CC02 (2021; citation chasing)	Other methods
T13	Shafabakhsh et al. 2020 – hemodialysis [29]	1	CC03	2020	CC03 (2020; citation chasing)	Other methods

The 23 included reports map to 13 independent randomized trial families. Publication counts are never used as independent-study counts in quantitative synthesis. Reference numbers in the anchor column correspond to the main manuscript.

Table S4. Characteristics of the 13 independent randomized controlled trials

Trial ID	Trial / reference	Country	Population	Intervention and dose	Duration	Comparator	Sample I/C*	Inflammatory outcome role
T01	Yaikwawong / TCTR20140303003 [31,45,52]	Thailand	Adults with T2DM and obesity; linked MASLD subset reports	Curcuminoid extract, 1500 mg/day	12 months	Placebo	113/114	hs-CRP, TNF-α quantitative; IL-6 and IL-1β narrative
T02	Asghari et al. 2024 [32]	Iran	Adults with T2DM	Nano-curcumin 80 mg/day + omega-3 placebos	12 weeks	Double placebo	24/23	hs-CRP quantitative
T03	El-Rakabawy et al. 2025 [33]	Egypt	T2DM with elevated ASCVD risk	Curcumin 1500 mg/day + conventional therapy	14 weeks	Conventional therapy	36/36	TNF-α quantitative
T04	Nada et al. 2025 [34]	Egypt	Uncontrolled T2DM with hyperlipidemia	Curcumin 1100 mg + black pepper 5 mg/day + glimepiride	12 weeks	Placebo + glimepiride	20/20	hs-CRP quantitative after median/IQR conversion; eligible contrast used curcumin + glimepiride vs placebo + glimepiride; fenofibrate + glimepiride arm excluded
T05	Dastani et al. 2023 [35]	Iran	T2DM + mild-to-moderate coronary artery disease	Nanomicellar curcumin 80 mg/day	90 days	Placebo	32/32	hs-CRP quantitative
T06	Mansour et al. 2025 [27]	Iran	T2DM + diabetic peripheral neuropathy	Nanocurcumin 80 mg/day	16 weeks	Placebo	41/45 analyzed	hs-CRP exploratory sensitivity only
T07	Usharani et al. 2008 [36]	India	Adults with T2DM	NCB-02 standardized curcuminoids 600 mg/day	8 weeks	Placebo	23/21	TNF-α quantitative; IL-6 narrative (group-level values reported); atorvastatin arm excluded
T08	Na et al. 2013/2014 [37,38]	China	Adults with T2DM	Curcuminoids 300 mg/day	3 months	Placebo	50/50	TNF-α quantitative; IL-6 narrative (group-level follow-up values reported; ANCOVA-adjusted inference); 2013/2014 reports mapped to one trial
T09	Panahi / IRCT201505301165N4 [28,39]	Iran	Adults with T2DM	Curcuminoids 500 mg/day + piperine 5 mg/day	12 weeks	Placebo	50/50 completed	hs-CRP SMD sensitivity; TNF-α narrative; linked reports counted once
T10	Adibian / Hodaei cohort [40]	Iran	Adults with T2DM	Curcumin 1500 mg/day	10 weeks	Placebo	21/23	hs-CRP quantitative
T11	Abbood et al. 2018 [41]	Iraq	Poorly controlled T2DM + dyslipidemia	Curcumin 1000 mg/day	12 weeks	Placebo	16/16	TNF-α quantitative; atorvastatin arm excluded
T12	Mokhtari et al. 2021 [42]	Iran	T2DM + grade 3 diabetic foot ulcer	Nanocurcumin 80 mg/day	12 weeks	Placebo	25/25	hs-CRP quantitative
T13	Shafabakhsh et al. 2020 – hemodialysis [29]	Iran	T2DM on maintenance hemodialysis	Nanocurcumin 80 mg/day	12 weeks	Placebo	30/30 randomized	hs-CRP adjusted-effect sensitivity only

**Sample sizes are analyzed participants for the relevant comparison where available; T13 is reported as randomized 30/30 because compatible group-level hs-CRP endpoint data were not verified for the primary MD synthesis. In multi-arm trials, the single eligible contrast isolating curcumin/curcuminoids against placebo or standard care with equivalent background therapy was extracted; additional active-comparator or non-isolating combination arms were excluded, and no shared control group was entered more than once within a synthesis. For T09, the dose shown follows the 2018 inflammatory-biomarker report used for this review (curcuminoids 500 mg/day plus piperine 5 mg/day); linked publications from the same registered trial family reported different nominal doses, so outcome-specific report details were retained in the extraction audit. For Nada et al. [34], the eligible quantitative comparison was curcumin + glimepiride versus placebo + glimepiride; the fenofibrate + glimepiride arm was excluded.*

Table S5. Outcome-level extraction, data preparation, and analytical role

Panel A. hs-CRP extraction and preparation decisions.

Trial	n I/C	Intervention endpoint	Control endpoint	Preparation	Analysis-ready effect / audit detail	Role
T05 Dastani 2023	32/32	1.48 ± 0.96	2.05 ± 1.79	Direct mean ± SD	MD = -0.57; SE = 0.3591	Primary MD
T04 Nada 2025	20/20	7.85 (Q1 5.33; Q3 9.79)	11.65 (Q1 6.50; Q3 15.65)	Wan conversion: 7.6567 ± 3.5584	Control: 11.2667 ± 7.3003; MD = -3.61; SE = 1.8160; eligible contrast: curcumin + glimepiride vs placebo + glimepiride; fenofibrate arm excluded	Primary MD; fenofibrate arm excluded
T02 Asghari 2024	24/23	7.31 ± 2.21	9.11 ± 2.55	Direct mean ± SD	MD = -1.80; SE = 0.6973	Primary MD
T01 Yaikwawong 2024	113/114	2.60 [min–max 0.14–14.60]	3.75 [min–max 0.25–30.24]	Published means retained; SD from range (Wan): 2.8448 / 5.8929	MD = -1.15; SE = 0.6134	Primary MD
T12 Mokhtari 2021	25/25	15.4 ± 16.3	22.1 ± 17.5	Direct mean ± SD	MD = -6.70; SE = 4.7831; adjusted β = -4.97 (95% CI -12.27 to 2.32; p=0.17)	Primary MD
T10 Adibian 2019	21/23	2.9 ± 2.9	3.4 ± 4.2	Direct mean ± SD	MD = -0.50; SE = 1.0805	Primary MD
T09 Panahi 2017/2018	50/50	Published hs-CRP values with physiologically implausible printed unit	Same report	Do not force conversion to mg/L	Unit-invariant standardized effect used	SMD sensitivity only
T13 Shafabakhsh HD 2020	30/30 randomized	Compatible group-level endpoint means/SD not verified	—	Baseline-adjusted effect retained separately	β = -0.78 mg/L (95% CI -1.41 to -0.15); derived SE ≈ 0.3214	Adjusted-effect sensitivity only
T06 Mansour 2025	41/45	Final 1.80 (Q1 1.11; Q3 4.10)	Final 1.15 (Q1 0.60; Q3 2.30)	Baseline imbalance; trial used adjusted ANCOVA	Adjusted p=0.217; treatment effect and SE not reported	Exploratory; not pooled

All hs-CRP values are treated in mg/L when used in the primary MD synthesis. Wan et al. [26] was used only where endpoint dispersion was not directly reported. Panahi was retained in a unit-invariant SMD sensitivity analysis because the printed hs-CRP unit was physiologically implausible and could not be reliably verified; no unverified unit conversion was assumed. The Shafabakhsh-HD effect was kept separate because it is a baseline-adjusted estimate. Mansour was not quantitatively combined because the adjusted ANCOVA treatment effect and standard error were not reported.

Panel B. TNF-α extraction and preparation decisions.

Trial	n I/C	Intervention endpoint	Control endpoint	Preparation	Effect used	Role
T01 Yaikwawong 2024	113/114	3.46 [min–max 1.33–8.59]	6.77 [min–max 2.14–15.37]	Mean retained; SD from range	Hedges g = -1.571; 95% CI -1.869 to -1.273	Primary SMD
T03 El-Rakabawy 2025	36/36	16.5 ± 7.4	20.6 ± 5.7	Direct mean ± SD	Hedges g = -0.614; 95% CI -1.087 to -0.141	Primary SMD
T07 Usharani 2008	23/21	1.45 ± 1.20	3.96 ± 1.37	Direct mean ± SD	Hedges g = -1.920; 95% CI -2.640 to -1.200	Primary SMD
T08 Na 2013/2014	50/50	1.72 ± 0.74	2.14 ± 0.84	Follow-up mean ± SD from linked secondary inflammatory report (letter format) containing original randomized-trial data	Hedges g = -0.527; 95% CI -0.925 to -0.128	Primary SMD
T11 Abbood 2018	16/16	56.85 ± 5.73	69.46 ± 9.42	Direct mean ± SD	Hedges g = -1.577; 95% CI -2.376 to -0.777	Primary SMD
T09 Panahi 2017/2018	50/50 completed	TNF-α measured; group-level endpoint mean/SD unavailable	—	No auditable SMD could be derived	Between-group reduction reported (p<0.001)	Narrative only

TNF-α was synthesized with Hedges' g because outcome distributions and effective scales differed materially across trials despite nominally similar units. Negative standardized effects favor curcumin.

Panel C. IL-6 and IL-1β narrative evidence.

Biomarker	Trial	n I/C	Intervention endpoint	Control endpoint	Final synthesis role
IL-6	T01 Yaikwawong 2024	113/114	6.12 [3.09–12.40]	15.84 [4.33–19.66]	Narrative synthesis; group-level numerical values reported; not pooled in the final analysis.
IL-6	T07 Usharani 2008	23/21	1.83 ± 0.67	3.57 ± 2.21	Narrative synthesis; group-level mean ± SD values reported; not pooled in the final analysis.
IL-6	T08 Na 2013/2014	50/50	2.68 ± 1.63	3.90 ± 2.04	Narrative synthesis; group-level follow-up mean ± SD values reported. Between-group follow-up inference in the linked secondary report used ANCOVA adjusted for baseline values and covariates; not pooled in the final analysis.
IL-1β	T01 Yaikwawong 2024	113/114	0.31 [0.10–1.39]	0.74 [0.32–1.86]	Narrative; only one independent randomized cohort

Group-level numerical IL-6 values were reported for three independent randomized cohorts (T01, T07, and T08). IL-6 was nevertheless retained as a narrative outcome because only three trials contributed and there were substantial differences in intervention duration, formulation, outcome distribution/reporting, and analytical approach, including ANCOVA-adjusted between-group inference in the Na secondary report. The designation “narrative” refers to the final synthesis role, not to the absence of numerical outcome data. IL-1β was available from only one independent randomized cohort.

Table S6. Detailed result-specific Cochrane RoB 2 assessment

Panel A. Consensus domain judgments for the 13 included trial families.

Trial	Study	Assessed inflammatory result	D1	D2	D3	D4	D5	Overall
T01	Yaikwawong / TCTR20140303003 [31,45,52]	hs-CRP primary; TNF-α also quantified	Low	Low	Low	Low	Some concerns	Some concerns
T02	Asghari et al. 2024 [32]	hs-CRP	Low	Low	Low	Low	Some concerns	Some concerns
T03	El-Rakabawy et al. 2025 [33]	TNF-α	Some concerns	Some concerns	Some concerns	Low	Some concerns	Some concerns
T04	Nada et al. 2025 [34]	hs-CRP	Some concerns	Low	Some concerns	Low	Some concerns	Some concerns
T05	Dastani et al. 2023 [35]	hs-CRP	Some concerns	Low	Low	Low	Some concerns	Some concerns
T06	Mansour et al. 2025 [27]	hs-CRP	Low	Low	Some concerns	Low	Some concerns	Some concerns
T07	Usharani et al. 2008 [36]	TNF-α primary; IL-6 also reported	Some concerns	Some concerns	Some concerns	Low	Some concerns	Some concerns
T08	Na et al. 2013/2014 [37,38]	TNF-α primary; IL-6 secondary	Some concerns	Low	Low	Low	Some concerns	Some concerns
T09	Panahi / IRCT201505301165N4 [28,39]	hs-CRP sensitivity; TNF-α narrative	Some concerns	Low	Some concerns	Low	Some concerns	Some concerns
T10	Adibian / Hodaei cohort [40]	hs-CRP	Some concerns	Low	Some concerns	Low	Some concerns	Some concerns
T11	Abbood et al. 2018 [41]	TNF-α	Some concerns	Some concerns	Low	Low	Some concerns	Some concerns
T12	Mokhtari et al. 2021 [42]	hs-CRP	Some concerns	Low	Some concerns	Low	Some concerns	Some concerns
T13	Shafabakhsh et al. 2020 – hemodialysis [29]	hs-CRP adjusted effect	Some concerns	Low	Some concerns	Low	Some concerns	Some concerns

D1, bias arising from the randomization process; D2, bias due to deviations from intended interventions; D3, bias due to missing outcome data; D4, bias in measurement of the outcome; D5, bias in selection of the reported result. All 13 included trial-family rows were judged as having some concerns overall; none was judged at high overall risk. R0837/T14 is excluded from the final review and is not displayed.

Panel B. Concise audit rationale supporting the consensus judgments.

Trial	Study	Assessed result	Audit rationale
T01	Yaikwawong / TCTR20140303003 [31,45,52]	hs-CRP primary; TNF-α also quantified	Adequate randomization/concealment and double blinding; complete outcome data and objective laboratory measurement. D5 retained as some concerns because hs-CRP was not explicitly specified as a detailed registry/SAP outcome.
T02	Asghari et al. 2024 [32]	hs-CRP	Computer/block randomization with opaque envelopes, blinding, minimal attrition, and objective hs-CRP measurement. D5: registry outcome specification was broad and no detailed SAP was verified.
T03	El-Rakabawy et al. 2025 [33]	TNF-α	Open-label trial; sequence generation/concealment incompletely described and 72/85 randomized participants were analyzed. TNF-α was an objective laboratory outcome. D5: no detailed prospective reporting plan verified.
T04	Nada et al. 2025 [34]	hs-CRP	Computer-generated sequence but concealment not explicit; double-blind; 60/67 participants completed the three-arm trial. Objective hs-CRP measurement. D5: incomplete prospective hs-CRP reporting specification.
T05	Dastani et al. 2023 [35]	hs-CRP	Sequence generation/concealment details were insufficiently reported; double-blind with no material missing outcome issue and objective hs-CRP measurement. D5: no prospectively verified detailed SAP.
T06	Mansour et al. 2025 [27]	hs-CRP	Block randomization/concealment and blinding were adequate. Attrition occurred (86/97 completed) and analysis/reporting did not fully support an ITT interpretation. D5: adjusted ANCOVA effect and SE were not reported and no detailed SAP was verified.
T07	Usharani et al. 2008 [36]	TNF-α primary; IL-6 also reported	Randomization/concealment and blinding procedures were incompletely described; 67/72 participants completed the trial. TNF-α/IL-6 were objective laboratory outcomes. D5: no verified registry/SAP.
T08	Na et al. 2013/2014 [37,38]	TNF-α primary; IL-6 secondary	Block randomization described but concealment not reported; double-blind and complete analyzed sample. Registry timing/outcome specification did not clearly prespecify TNF-α/IL-6; D5 therefore some concerns.
T09	Panahi / IRCT201505301165N4 [28,39]	hs-CRP sensitivity; TNF-α narrative	Randomization/concealment insufficiently detailed; double-blind; 100/118 completed with broadly balanced attrition. Objective biomarker measurement, but hs-CRP unit ambiguity and lack of a detailed SAP support D5 some concerns.
T10	Adibian / Hodaei cohort [40]	hs-CRP	Sex-stratified block randomization reported but concealment not explicit; double-blind; 44/53 participants analyzed. CRP was registered, but a full prespecified analysis plan was not verified.
T11	Abbood et al. 2018 [41]	TNF-α	Random assignment reported without details of sequence generation/concealment; blinding unclear. No material post-randomization TNF-α missing-data problem was identified and ELISA measurement was objective. No registry/SAP was verified.
T12	Mokhtari et al. 2021 [42]	hs-CRP	Randomization reported but sequence/concealment insufficiently detailed; double-blind; 50/60 analyzed. Objective hs-CRP measurement. hs-CRP

Trial	Study	Assessed result	Audit rationale
			was secondary and no detailed registry/SAP was verified.
T13	Shafabakhsh et al. 2020 – hemodialysis [29]	hs-CRP adjusted effect	Randomization reported but sequence/concealment insufficiently detailed; double-blind. Approximately 53/60 participants were analyzed and detailed missing-data sensitivity was not verified. Objective hs-CRP; prospective specification of the adjusted estimand was not verified.

The rationales summarize the locked reviewer consensus evidence without converting RoB 2 into a generic study-quality score; the judgments pertain to the inflammatory result used in this review.

Table S7. GRADE evidence profile for the primary quantitative outcomes

Outcome	RCTs	Participants	Primary effect	Risk of bias	Inconsistency	Indirectness	Imprecision	Publication bias	Certainty
hs-CRP	6	472	MD -1.06 mg/L (95% CI -1.97 to -0.16)	Serious (-1)	Not serious; I^2=17.3%	Not serious	Not serious for prespecified primary estimate; six compatible endpoint contrasts and 95% CI excluded null. Sensitivity analyses with alternative data handling reduced precision and were treated as robustness limitations.	Not downgraded; formal asymmetry testing not feasible (<10 trials)	Moderate (⊕⊕⊕○)
TNF-α	5 (+1 narrative)	475	Hedges g -1.20 (95% CI -1.98 to -0.42)	Serious (-1)	Serious (-1); I^2=84.9%	Not serious	Not serious for primary estimate	Not downgraded; formal asymmetry testing not feasible (<10 trials)	Low (⊕⊕○○)

RCT evidence started at high certainty. hs-CRP was downgraded one level for risk of bias. Imprecision was not downgraded for the prespecified primary hs-CRP estimate because six compatible endpoint contrasts contributed and the 95% CI excluded the null; however, alternative data-handling sensitivity analyses reduced precision and were treated as important robustness limitations in the interpretation. TNF-α was downgraded one level for risk of bias and one level for serious inconsistency. Publication bias was not downgraded for either outcome because no specific evidence was identified, although formal asymmetry testing was not informative with fewer than ten trials.

Table S8. Sensitivity, subgroup, and leave-one-out analyses

Panel A. hs-CRP primary and prespecified/exploratory sensitivity analyses.

Analysis	Studies	Participants	Effect (95% CI)	p	I²	Interpretation
hs-CRP primary REML–Hartung–Knapp	6	472	MD -1.06 mg/L (-1.97 to -0.16)	0.030	17.3%	Primary inception analysis
hs-CRP REML–Wald sensitivity	6	472	MD -1.06 mg/L (-1.73 to -0.40)	0.002	17.3%	Same point estimate; narrower interval
hs-CRP direct endpoint mean/SD only	4	205	MD -0.95 mg/L (-2.36 to 0.45)	0.120	24.0%	Direction unchanged; greater imprecision
hs-CRP unit-invariant SMD including Panahi	7	572	Hedges g -0.28 (-0.58 to 0.02)	0.062	46.1%	Direction favorable; CI crosses null
hs-CRP GIV including Shafabakhsh-HD adjusted effect	6 unadjusted + 1 adjusted	Mixed estimands	MD ≈ -0.88 mg/L (-1.41 to -0.35)	0.007	0%	Exploratory only; adjusted and unadjusted estimands combined
Mansour adjusted ANCOVA	1	86 analyzed	Not estimable for pooling	0.217 (adjusted comparison)	—	Adjusted treatment effect and SE were not reported; retained as exploratory only

For the Shafabakhsh-HD generic inverse-variance sensitivity, the pooled result mixes adjusted and unadjusted estimands and therefore does not replace the primary endpoint MD synthesis. Mansour could not enter an adjusted-effect meta-analysis because the adjusted effect estimate and standard error were unavailable.

Panel B. hs-CRP leave-one-out analysis from the final six-RCT primary dataset.

Excluded trial	Pooled MD (95% CI)	p (HK)	I²	τ² (REML)	Interpretation
Dastani et al. 2023	-1.45 (-2.57 to -0.32)	0.023	0%	0.000	Effect remains favorable; heterogeneity falls to 0%
Nada et al. 2025	-0.94 (-1.79 to -0.10)	0.036	11.2%	0.063	Direction and significance retained
Asghari et al. 2024	-0.80 (-1.69 to 0.09)	0.066	0%	0.000	Direction retained; CI crosses null
Yaikwawong et al. 2024	-1.17 (-2.59 to 0.26)	0.086	31.3%	0.358	Direction retained; largest residual heterogeneity
Mokhtari et al. 2021	-1.02 (-1.96 to -0.09)	0.038	18.1%	0.106	Effect remains favorable
Adibian et al. 2019	-1.19 (-2.40 to 0.01)	0.051	30.6%	0.243	Returns prior five-trial borderline HK result

Panel C. TNF-α primary and Wald sensitivity analyses.

Analysis	Studies	Participants	Effect (95% CI)	p	I²	Interpretation
TNF-α primary REML–Hartung–Knapp	5	475	Hedges g -1.20 (-1.98 to -0.42)	0.013	84.9%	Primary quantitative synthesis
TNF-α REML–Wald sensitivity	5	475	Hedges g -1.20 (-1.76 to -0.65)	<0.001	84.9%	Same direction; narrower interval

Panel D. TNF-α leave-one-out analysis.

Excluded trial	Pooled Hedges g (95% CI)	p (HK)	I^2	τ^2 (REML)	Interpretation
Yaikwawong et al. 2024	-1.10 (-2.20 to 0.004)	0.050	82.7%	0.386	Direction unchanged; significance becomes borderline
El-Rakabawy et al. 2025	-1.36 (-2.34 to -0.38)	0.022	84.1%	0.320	Direction unchanged; substantial heterogeneity persists
Usharani et al. 2008	-1.05 (-1.97 to -0.13)	0.036	84.8%	0.280	Direction unchanged; substantial heterogeneity persists
Na et al. 2013/2014	-1.39 (-2.29 to -0.48)	0.016	78.4%	0.251	Direction unchanged; heterogeneity remains substantial
Abbood et al. 2018	-1.13 (-2.22 to -0.05)	0.045	89.0%	0.393	Direction unchanged; substantial heterogeneity persists

Negative MDs and Hedges' g values favor curcumin. I^2 values are those returned by metafor using its general random-effects definition based on τ^2 and the typical within-study sampling variance; they may therefore differ from the simple Q-based approximation. Across TNF-α leave-one-out analyses, the direction of effect remained favorable, but substantial heterogeneity persisted. The hs-CRP leave-one-out analysis similarly preserved a negative pooled direction in every iteration, although confidence intervals crossed the null for some exclusions. Panel E reports exploratory formulation/duration subgroup analyses requested during peer review.

Panel E. Exploratory subgroup analyses requested during peer review.

Subgroup estimates use separate REML random-effects models with Hartung-Knapp confidence intervals. Tests of subgroup differences use mixed-effects REML meta-regression with Hartung-Knapp inference. Duration was categorized as ≤12 weeks versus >12 weeks; the 90-day Dastani trial was classified as >12 weeks. A TNF-α formulation subgroup was not estimable because all five quantitative TNF-α trials used conventional/standardized non-nano preparations. These analyses are exploratory because each subgroup contains only two to four trials.

Outcome	Factor	Subgroup	RCTs	Participants	Effect (95% CI)	I²	Pinteraction	Interpretation
hs-CRP	Formulation	Nano/nanomicellar	3	161	MD -1.14 mg/L (-3.99 to 1.70)	46.2%	0.849	No detectable subgroup difference; underpowered
hs-CRP	Formulation	Conventional/standardized	3	311	MD -1.20 mg/L (-3.50 to 1.10)	0%	0.849	No detectable subgroup difference; underpowered
hs-CRP	Duration	≤12 weeks	4	181	MD -1.69 mg/L (-3.58 to 0.20)	0%	0.205	No detectable subgroup difference; underpowered
hs-CRP	Duration	>12 weeks	2	291	MD -0.72 mg/L (-3.93 to 2.50)	0%	0.205	No detectable subgroup difference; underpowered
TNF-α	Duration	≤12 weeks	3	176	Hedges' g -1.29 (-3.14 to 0.56)	82.5%	0.800	No detectable subgroup difference; high heterogeneity
TNF-α	Duration	>12 weeks	2	299	Hedges' g -1.11 (-7.19 to 4.96)	91.1%	0.800	No detectable subgroup difference; high heterogeneity

Pinteraction values are from mixed-effects meta-regression. Lack of a statistically detectable interaction should not be interpreted as equivalence across formulations or durations because the subgroup analyses are severely underpowered.

Audit-source files used for Tables S2–S8: Curcumin_T2DM_human_screening_Reviewer1and2_COMPLETE_01Sep2026.xlsx; Curcumin_T2DM_PRISMA_CLOSEOUT_01Sep2026.xlsx; Curcumin_T2DM_Audited_Master_Extraction.xlsx; Curcumin_T2DM_hsCRP_FINAL_recalculated_from_master_31Aug2026.xlsx; Curcumin_T2DM_TNFalpha_master_audit_31Aug2026.xlsx; Curcumin_T2DM_RoB2_CONSENSUS_FINAL_14_trials_01Sep2026.xlsx. Final eligibility, the 2026 Yaikwawong linked report, and the final six-RCT hs-CRP estimates were reconciled to the submission-ready manuscript dated 6 September 2026.
